# Assessing the Influence of Selected Permeabilization Methods on Lymphocyte Single-Cell Multi-Omics

**DOI:** 10.3390/antib14010015

**Published:** 2025-02-10

**Authors:** Shifan Ding, Na Lu, Hassan Abolhassani

**Affiliations:** 1 Division of Immunology, Department of Medical Biochemistry and Biophysics, Karolinska Institute, 17177 Stockholm, Sweden; dingsh@gmail.com (S.D.); lu.na@gmail.com (N.L.); 2 Research Center for Immunodeficiencies, Pediatrics Center of Excellence, Children’s Medical Center, Tehran University of Medical Sciences, Tehran 1419733151, Iran

**Keywords:** lymphocytes, single-cell sequencing, multi-omics, transcriptome, proteome

## Abstract

(1) Background: Single-cell multi-omics is a powerful method for the dissection and detection of complicated immunologic functions and synapses. However, most currently available technologies merge datasets of different omics from separate portions of the same sample to generate combined multi-omics. This process is a source of bias, mainly in the field of immunology on cells originating from pluripotent hematopoietic stem cells with high flexibility during maturation. (2) Methods: Although new multi-omics approaches have been developed to use the advantages of cellular and molecular barcoding and next-generation sequencing to solve this issue, one of the main current challenges is intracellular proteomics, which should be combined with other omics data with high importance for immune system studies. We designed this study to evaluate previously recommended minimal permeabilization and fixation methods on the quality and quantity of transcriptomics and proteomics data generated by the BD Rhapsody™ Single-Cell Analysis System. (3) Results: Our findings showed that high-throughput sequencing with advanced quality and read-out is required for the combination of multi-omics outcomes from a permeabilized single cell. Therefore, the HiseqX platform was selected for further analysis. The effect of immune stimulation was observed clearly as the separated clusters of helper and cytotoxic T cells using unsupervised clustering. Importantly, fixation and permeabilization did not affect the general expression profile of unstimulated cells. However, fixation and permeabilization were proved to negatively impact the detection of the whole transcriptome for single-cell assay. Nevertheless, about 60% of the transcriptomic signature of the stimulation was detected. If the measurement of combined surface and intracellular markers is required to be achieved, the modified fixation and permeabilization method is recommended because of a lower transcriptomic loss and more precise proteomic fingerprint detected. (4) Conclusions: The findings of this study support the potential possibility for integrating intracellular proteomics, which needs to be optimized and tested with newly designed oligonucleotide-tagged antibodies targeting intracellular proteins.

## 1. Introduction

Lymphocytes are the main biological units responsible for forming multicellular adaptive immunity. The intrinsic phenotypic transformations in lymphocytes occur at single-cell resolution. The single-cell multi-omics approach is a method that can detect lymphocyte characteristics at an individual cell level. Both transcriptomic and proteomic assays have been used to reveal the molecular expression and interactions within single lymphocytes under different conditions [1]. Generally, expression data for individual cell types can be obtained by labeling specific markers to identify the lymphocyte entities [2,3]. Variable methods have been developed since the introduction of single-cell transcriptomics data produced by the micro-pipetting of single cells [4]. Moreover, fluorescent-activated cell sorting (FACS) combined with microdroplets or microfluidic chips has been used more commonly for selecting single cells [2]. Besides transcriptome, the corresponding proteomics profile can be explored simultaneously by tagging specific antibodies on proteins of interest [5]. An improved method called mass cytometry, using metal isotopes as reporters, can measure more cellular parameters in individual cells but still provide only one omics dimension [6,7]. Instead of detecting mass or fluorescence reporters, stained cells by polyadenylic acid oligonucleotide barcoded antibodies (Oligo-Ab) can also profile and combine the targeted proteomics while performing the single-cell transcriptomics protocols simultaneously [8].

Because of the power of the single-cell multi-omics method, it has been proposed to apply to the investigation of the immune system in various physiological and pathological conditions [9,10,11]. Indeed, transcriptomics and proteomics analyses are the main driving omics since they provide a clue for the interpretation of either regulatory genetic/epigenetic omics (DNA mutations and modifications, histone modifications, genome structure and availability, miRNA profiles, etc.) or consequential intra- or extracellular functional omics (interactome, metabolome, immunome, etc.) [12,13]. Integrating both transcriptomics and proteomics aspects into one measurement of the single-cell multi-omics method can provide a further understanding of the linkage between RNA expression level and phenotypic cellular state at one immune cell resolution [14].

Until now, most developed technologies have focused on the combination of transcriptomics with membrane/surface proteomics but not intracellular proteomics. The main barrier to integrating intracellular protein assays is the impact of fixation and permeabilization (required for intracellular antibody staining) on reducing and losing RNA integrity [15,16]. This pitfall is very crucial to be solved before emerging other omics-layers for immune functional assay. Intracellular proteomics should be available from the same single cell to measure the responses of lymphocytes when certain stimulations are given [17]. The recently introduced method of intracellular staining and sequencing (INs-seq) of fixed and permeabilized immune cells is limited by fluorophore-conjugated antibodies and the sorting of fluorescence-activated cells [18]. Moreover, the effect of fixation and permeabilization on the transcriptomics quality has not been optimized using a standard multi-omics method. So, to extend the scale of proteomics analysis other than the surface marker labeling and explore more opportunities for single-cell multi-omics sequencing, the assessments of fixation and permeabilization methods on lymphocytes were performed in this study. We evaluated its influences on transcriptomic profiling for single-cells captured by the BD Rhapsody™ Single-Cell analysis-based method.

## 2. Materials and Methods

### 2.1. Design of the Study

To evaluate the impact of fixation (preserving the states of the cells under the controlled stimulated conditions and stopping all the enzyme activities) and permeabilization (opening cellular membranes for accessing intracellular proteins) [19,20], we designed the following new approach. Compared to the common method of cell sorting and capturing single cells, the BD Rhapsody™ Single-Cell analysis-based method, “CytoSeq”, has been developed for measuring mRNA and protein expression levels for individual cells more precisely and more abundantly with fewer cell manipulation steps [21]. Unlike classifying the cells by fluorescent markers [22], this method uses small pico-liter wells for single-cell deposition, and within each well, there is a magnetic bead, which contains cell barcoding and capturing probes for RNA and attached Oligo-Ab. Therefore, this method can acquire transcriptomic and proteomic information for an individual immune cell [21]. We aimed to evaluate the impact of fixation and permeabilization on this multi-omics technology for immunologic studies.

### 2.2. Isolation and Processing of Peripheral Blood Mononuclear Cells (PBMCs)

The study has approval from the Karolinska Institute’s ethical committee. Blood samples from healthy individuals have been collected after obtaining written informed consent. An amount of 4 mL of Ficoll-Paque PLUS solution (Lymphoprep™, STEMCELL Technologies, Vancouver, BC, Canada) was added to about 10 mL of peripheral blood and then centrifuged for 30 min at 1700 rpm. PBMCs were collected and then frozen in commercial freezing media (Gibco™, Synth-a-Freeze™ Cryopreservation Medium, Waltham, MA, USA) at a cell concentration of 5 × 10^6^ cells/µL in the liquid nitrogen. PBMCs were thawed during the experiment and transferred to a 37 °C Roswell Park Memorial Institute medium (RPMI 1640 Complete Medium, with 10% fetal bovine serum, 0.5% penicillin–streptomycin, Gibco™, Grand Island, NY, USA).

### 2.3. Stimulation, Fixation, and Permeabilization

**Figure 1:**
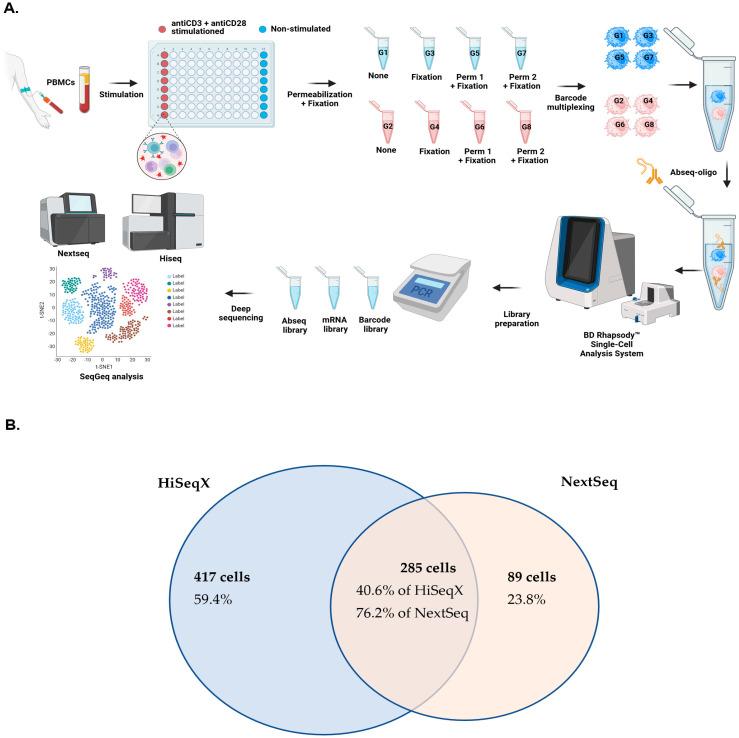
(**A**) Overview of the methods and the study design used to evaluate the impact of fixation and permeabilization on single-cell multi-omics BD Rhapsody assays. (**B**) Operation of NextSeq (by read length 2 × 150 bp, output ≥ 90 Gb) and HiSeqX datasets (read length 2 × 150 bp, output ≥ 1.6 Tb) and the detected unique qualified cells and overlapping number of cells based on the unique cell barcodes. Percentages are calculated based on the cells detected in the same color clusters of Nextseq (pink circle) or HiseqX (blue circle).

Eight different conditions were designed for control and comparison (Table 1, Figure 1A). Immune stimulation was performed in clear flat-bottom Immuno nonsterile 96-well plates (Thermo Fisher, Waltham, MA, USA). Anti-CD3 (clone: OKT3, Invitrogen, Waltham, MA, USA) and anti-CD28 (clone: NA/LE, BD Pharmingen, San Jose, CA, USA) antibodies were coated on the plate overnight at 37 °C, with a concentration of 1 mg/well. Then, cells were seeded in the antibodies-coated (labeled as stimulated) and non-coated (unstimulated cells) wells for 24 h at 37 °C, with a cell concentration of 1 × 10^5^ cells/well. Considering the cryopreservation of cells and the possibility of increased fragile cells prone to die, the viability of cells was controlled before the next step (~97%), and the same sample was used for technical replicates to avoid the impact of apoptosis or dead cells variation on the downstream single-cell multi-omics output. Fixation/Permeabilization method 1 is achieved using BD Cytofix/Cytoperm Buffer (following the manufacturer’s instructions, BD Biosciences, Oxford, UK), a selected method developed by BD company, which theoretically might be optimal for integration with BD Rhapsody™ Single-Cell Analysis System antibodies and reagents. According to this protocol, cells were resuspended thoroughly in 250 µL of the BD Fixation/Permeabilization solution for both stimulated and unstimulated cells for 20 min at 4 °C. The cells were washed twice in 1× BD Perm/Wash™ buffer (1 mL/wash, BD Biosciences) and pelleted. Another Fixation/Permeabilization method (method 2) was selected from the recommended method by Amidzadeh et al. [23] experiment, where they identified the best protocol of Fixation/Permeabilization with the lowest impact on the transcriptome profile. Briefly, the cells were fixed in 2% cold and freshly prepared paraformaldehyde in phosphate-buffered saline (PBS) and then permeabilized by 200 μL of Tween-20 at a concentration of 0.2%. Two sets of negative control groups without permeabilization, with and without fixation (by 2% paraformaldehyde), were also designed to compare the quality of the combined multi-omics outcome.

### 2.4. BD Rhapsody™ Single-Cell Analysis System Method

After the permeabilization/fixation step, an equal number of cells (3 × 10^5^ in 200 μL Stain Buffer, BD Pharmingen) from 8 different conditions were then labeled by various sample tags (BD Human Single-Cell Multiplexing Kit, BD, Table S1). Subsequently, all groups of cells were pooled into one main tube and were labeled by 2X BD Abseq-oligo Labeling Master-mix (2 μL of each Oligo-Ab in 100 μL of stain buffer, BD, Table S2) to decrease the chance of batch effect during antibody staining. Based on the official protocol applied, single-cell capture of the BD Rhapsody cartridge and cDNA synthesis parts was achieved by the BD Rhapsody Single-Cell Analysis System (BD). After the generation of the whole transcriptome and targeted proteome on the library beads, they were treated with exonuclease. Library preparation on Sample Tag (indicators of 8 desired conditions), Oligo-Ab (Table S2), and targeted mRNA panel (BD Rhapsody Immune response panel, BD) were performed using three separate lines of multiplex–polymerase chain reaction (PCR, BD targeted amplification kit, BD, Figure 1A) [1,10]. All conditions were generated at one experiment without replicates, and sequencing was performed from the same molecular pool using two different platforms (generating technical replicates).

### 2.5. Library Sequencing and Bioinformatic Analysis

Libraries were pooled according to the final concentrations (based on manufacture protocol by single 8base-pair index, Illumina i7) and then sequenced on both the Illumina Nextseq and HiSeq-X (150 bp paired-end sequencing, Table S3). Demultiplexing was performed and reads were assigned with both expected and “impossible” barcode combinations (sequences not matched with the unique molecular identifiers of capturing beads). Reads were mapped to the reference genome, and the number of read pairs aligned to the exonic region of each gene was counted. Raw data were evaluated by the Seven Bridges Genomics platform (BD Rhapsody™ WTA Analysis Pipeline) for standard quality control, and metrics of the analysis, including the counts of mRNA and Oligo-Ab for every single cell, were reported. Each captured cell was assigned to one of eight experimental conditions based on the detected sample tags. The expression quality and quantity between experimental conditions and identified supervised immune-cell subsets were compared by R Statistical Software (R version 3.6.3, CRAN) and SeqGeq software (version 1.6.0, FlowJo LLC). For all statistical analyses, multiple comparison corrections for both transcriptome and proteome were performed, and an adjusted *p*-value below 0.05 after Hochberg sequential correction was considered significant. For detailed bioinformatic analysis, please see the Supplementary Methods.

## 3. Results

### 3.1. Comparing the Captured and Qualified Single Cells Between NextSeq and HiSeqX

First, two different datasets that were achieved from two different sequencing platforms were compared (Table 2). Both datasets were processed by the first step of filtration, and the output matrix for NextSeq had 419 captured cells, while HiSeqX had a higher number of captured cells at 751. There were 2899 mRNAs detected (expressed at least once within a single cell) in NextSeq, whereas HiSeqX had 6893 unique identified mRNAs, more than two-fold compared with NextSeq.

Although there were 15 Oligo-Abs detected from cells analyzed in both platforms, the median and interquartile range (IQR) values for detected Oligo-Abs in HiSeqX were similar or slightly higher compared to the NextSeq (except CD127, Table 3). Two more parameters were used for the comparison of the two platforms: the median (IQR) of library size was 53 (32–88) in HiSeqX and 34 (27–51) in NextSeq; the median (IQR) of genes expressed in HiSeqX was 35 (24–56) but in NextSeq was 26 (22–37). Based on selecting the range of 2.5% to 97.5% for these two parameters, 702 qualified cells were left in the HiSeqX dataset, while only 374 cells remained in the NextSeq dataset. The visualization of the set operation for two datasets is shown in Figure 1. Altogether, 285 qualified cells were identified with both NextSeq and HiSeqX (overlapped cells in Figure 1B), with 40.6% intersection of total qualified cells captured by HiSeqX and 76.2% of total qualified cells captured by the NextSeq dataset.

The captured cells and qualified cells from both platforms were assigned to their own experimental groups according to the detected sample tags (Table 1). The percentage of non-qualified NextSeq filtration (2–25%) was much larger than HiSeqX (2.5–11.7%). Also, the number of qualified cells in each group within the HiSeqX data was more balanced compared with the NextSeq cell group distribution (Figure S1). Both datasets have a relatively low number of cells captured and detected within experiment group 6 (G6, stimulated + Fixation/Permeabilization method 1 group), and experiment group 1 (G1, unstimulated) has the lowest percentage of unqualified cells, but NextSeq captured much fewer cells even in G1 compared to HiSeqX. Based on the findings of the first step and comparison of two sequencing platforms, our results demonstrate that the HiSeqX dataset had higher quality and more abundant data information. Therefore, HiseqX was selected and used for the downstream analysis.

For a closer inspection of the experimental conditions of the chosen HiSeqX dataset, it should be noted that experimental G1 and G2, the basement unstimulated and stimulated control groups, had the highest number of cells captured and qualified. Experimental G6 (stimulated + fixation/permeabilization method 1 group) had the lowest remaining number of cells and was low quality, with a high percentage of cells being filtered out. Experimental G7 (unstimulated + fixation/permeabilization method 2) performed better in terms of permeabilization compared to method 1 since the quality control step, with only 2.5% of cells being filtered out. Chi-square test goodness of fit was applied for each of the experimental groups; *p*-values of the tests are shown in Table S4. The *p*-values lower than 0.05 indicate significant differences between these two experimental groups in terms of expected qualified cell numbers. Although many studies have shown that the general process of permeabilization and fixation cannot impact the antibody staining with conventional Abs regarding the proteomics analysis, we investigated whether the sample tags using the multiplexing kit can affect permeabilization and fixation. However, when the original raw data of samples from G1 and G3 were compared, there was no higher rate of unassigned cells due to the absence of sample tag (5.4% vs. 3.4%, respectively, *p* = 0.87).

**Figure 2:**
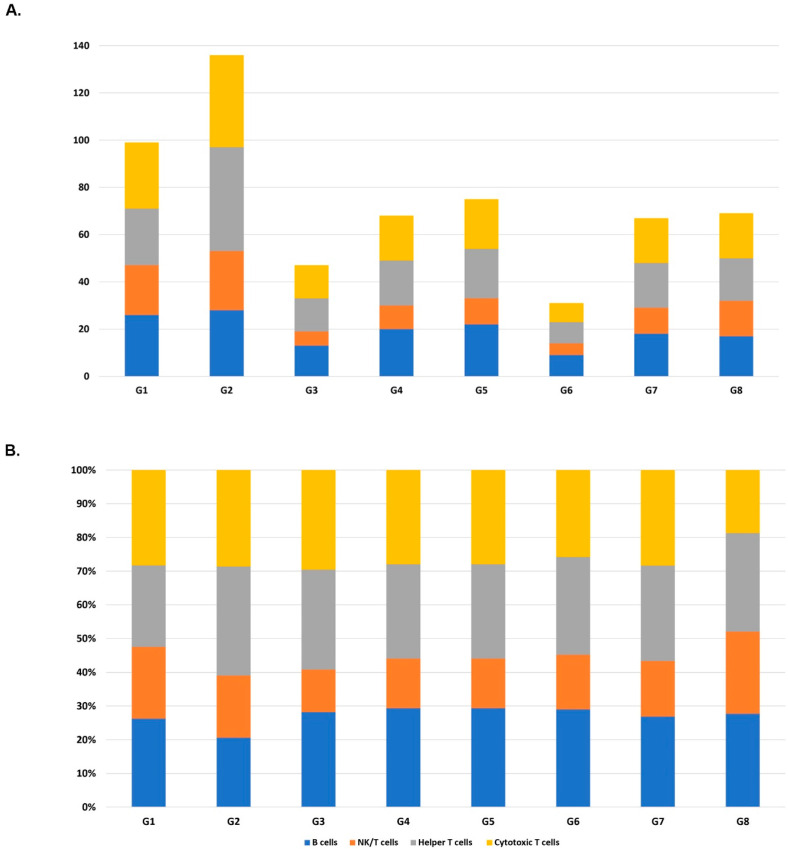
(**A**) Number of absolute lymphocyte counts for main immune subsets within 8 experimental groups and (**B**) the percentages of the cell types for that group. G1: Unstimulated PBMCs, G2: Stimulated PBMCs, G3: Unstimulated PBMCs with fixation, G4: Stimulated PBMCs with fixation, G5: Unstimulated PBMCs with Fixation/Permeabilization method 1, G6: Stimulated PBMCs with Fixation/Permeabilization method 1, G7: Unstimulated PBMCs with Fixation/Permeabilization method 2, and G8: Stimulated PBMCs with Fixation/Permeabilization method 2.

Regarding unsupervised clustering, a variable reduction step was then conducted. Of note, the median (IQR) of the cell-expressed parameter was 1 (1–3), and there were only 2459 mRNAs left after selecting the 2.5% to 97.5% of the cell-expressed density on the HiseqX dataset. Dispersions of these 2459 mRNAs were calculated, and 62 mRNAs were selected in the top 2.5% highly differentially expressed genes, as shown in Table S5. Supervised classification of detected populations of PBMCs was first defined based on their known immunological markers of proteomics and transcriptomic of G1 (Unstimulated group, Table S6). According to the immunological markers, the cells were assigned with their cell types in each group. The numbers of cells detected from each experimental condition and the ratios of the four cell types in the groups are summarized in Figure 2. Our results showed that percentages for each cell type among groups were generally balanced, supporting that the counts of main lymphocyte subsets were not affected significantly due to stimulation (G1 vs. G2), fixation (G1 vs. G3), and permeabilizations methods (G1 vs. G5 and G7; Table S7). Comparisons of G3 vs. G4, G5 vs. G6, and G7 vs. G8 assessing the influence of fixation or permeabilization on stimulation; of note, there was no significant difference between the number of detected cells in these cell types as well (Table S7).

### 3.2. Impact of Fixation and Permeabilization on Immune Stimulation Signature Detection Using BD Rhapsody

**Figure 3:**
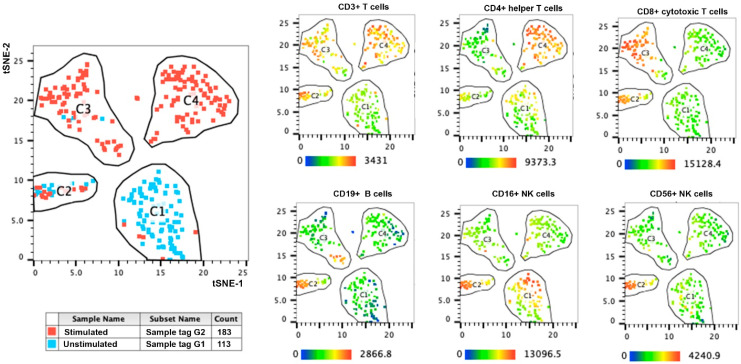
T-SNE plot of experimental conditions 1 and 2 based on the transformed expression of the differentially expressed markers in the cells from the PBMC dataset to detect the impact of stimulation. Then, manually added polygons in the t-SNE space were used for defining the main cell clusters. The blue color represents the cells from experimental condition G1 (unstimulated PBMCs without fixation and permeabilization), and the red color represents the cells from experimental condition G2 (stimulated PBMCs without fixation and permeabilization). Four main clusters can be detected between G1 and G2 conditions (labeled as C1–C4). Six main immune markers are depicted in the right panels to indicate the lymphocyte population changed due to anti-CD3/anti-CD28 stimulation between G1 and G2 experimental conditions within each newly detected clusters C1–C4. A range of unique sequenced UMI barcodes detected for each marker is shown as the key color legend.

The comparison of G1 and G2 was then conducted to observe the power of single-cell multi-omics for dissecting the effect of anti-CD3/anti-CD28 stimulation. As expected, the total number of cells detected for total T cells and the proportion of helper T cells were slightly increased after the stimulation (Figure 2). The t-SNE plot also depicted the separate clustering of stimulated helper and cytotoxic T cells versus the unstimulated PBMCs (Figure 3). Four clusters have been observed separating from each other and based on the expression pattern of cell markers shown in Figure 3, cluster 3 (C3) mainly contains the stimulated CD8 positive cells, and cluster 4 (C4) includes stimulated CD4 positive cells. The expressed mRNAs and surface proteins, which were significantly different between G1 and G2, are shown in Table S8.

Visualizations of the top significant parameters identified in each lymphocyte subset are depicted in Figure S2. Clusters 2, 3, and 4 were compared with the main cluster 1, tagged mainly by unstimulated cells. The volcano plots of differentially expressed parameters were supported by the observed significant variants between G1–G2 lymphocyte subsets (Figure S3). Then, we compared the effect of fixation and permeabilization of unstimulated cells by comparing G1 with G3 (fixation only, Figure S4), G5 (fixation-permeabilization method 1, Figure S5), and G7 (fixation-permeabilization method 2, Figure S6) experimental conditions. Although unsupervised clustering showed the absence of separation of clusters due to none of these methodological manipulations, the adverse impact of fixation and permeabilization method 2 was observed mainly on helper subsets and B cells in permeabilization method 1 (Figures S7–S9 and Tables S9–S11).

Since we observed that fixation and permeabilization did not meaningfully change the status of unstimulated cells, we moved to the next step to see if the fixed and permeabilized cell could reveal the observed transcriptomics and proteomics changes after stimulation between G1 vs. G2. Therefore, we first analyzed the expression profile between pairs of G3 vs. G4 (preservation of stimulation signature after fixation, Figures S11–S15, Table S12), G5 vs. G6 (preservation of stimulation signature after fixation-permeabilization method1, Figures S12 and S13, Table S13) and G7 vs. G8 (preservation of stimulation signature after fixation-permeabilization method2, Figures S14 and S15, Table S14). Comparing experiment pairs (G3–G4) (G5–G6) (G7–G8) vs. differentially expressed variants observed in (G1–G2) is shown as extracting the *t*-test *p*-values from each pair comparisons for those significant parameters observed in G1–G2 pair comparisons. The parameters were found significant while comparing groups 1 and 2 in helper T cells (Table S15), cytotoxic T cells (Table S16), and other lymphocyte subsets (Tables S17 and S18), which may not completely be recapitulated in any of the three other experimental pairs.

For helper T cells, only 10–14% of significant transcriptome fingerprint of stimulation was observed in permeabilized and fixed cells. However, significant proteomic fingerprint changes were documented in 46.1% of method 1 (none were significant) and 69.2% of method 2 (all were significant) permeabilization/fixation. Regarding cytotoxic T cells, a higher percentage of significant transcriptome fingerprints of stimulation were documented in permeabilized and fixed cells (16.6% vs. 30%). Similarly, a better outcome was achieved for the fingerprint of stimulation in proteomics data of method 1 (38.4%, which all were not significant) and method 2 (38.4%, which 60% of them were statistically significant).

**Figure 4:**
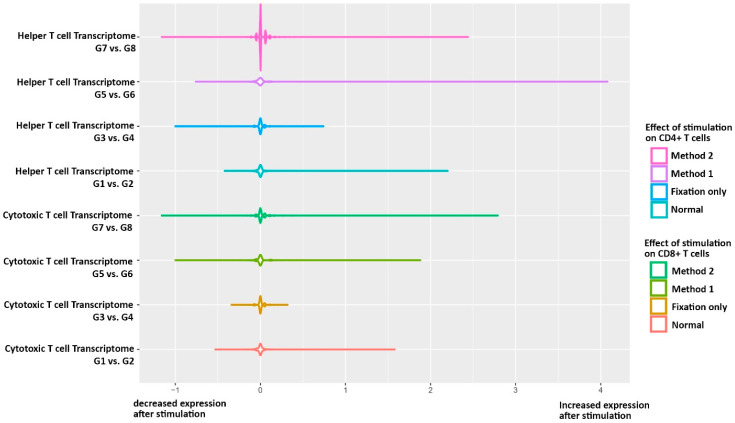
Violon plots of mRNA expression levels and pattern of distribution of targeted immune transcriptome within CD4+ helper T cells and CD8+ cytotoxic T cells among 8 different experimental groups before and after T cell stimulation via anti-CD3 and anti-CD8. The right lines (positive values) show the extent of unique genes increased after stimulation, and the left line (negative values) depicts the unique genes that decrease their expression after stimulation. G1: Unstimulated PBMCs, G2: Stimulated PBMCs, G3: Unstimulated PBMCs with fixation, G4: Stimulated PBMCs with fixation, G5: Unstimulated PBMCs with Fixation/Permeabilization method 1, G6: Stimulated PBMCs with Fixation/Permeabilization method 1, G7: Unstimulated PBMCs with Fixation/Permeabilization method 2, and G8: Stimulated PBMCs with Fixation/Permeabilization method 2.

The overall analysis of transcriptome data from helper T cells concludes that permeabilization/fixation methods 1 and 2 have similar preserved outcomes compared to the observed fingerprint of the stimulation (60.1% vs. 55.0% have a similar direction of changes, respectively). Interestingly, an overall analysis of transcriptome data from cytotoxic T cells showed that permeabilization/fixation methods 1 and 2 have similar preserved outcomes compared to the observed fingerprint of the stimulation (57.8% vs. 55.2% have a similar direction of changes, respectively, Figure 4). Considering condition G1 vs. G2 as the gold standard, we defined differentially expressed markers for each cell type subset to indicate the fraction of recovered differentially expressed genes in experimental conditions (G3 vs. G4, G5 vs. G6, and G7 vs. G8) in comparison with the gold standard (Table S19). We conclude that fixation and permeabilization, particularly method 2, did not meaningfully change the status of unstimulated cells and kept up to 60% of the signature of the immune stimulation at transcriptomics and proteomics levels.

## 4. Discussion

One of the main crucial steps in nucleotide barcoded multi-omics assays is the quality and quantity of the sequencing method. In this study, the same samples of purified library products were sequenced by two different platforms: HiSeq X and NextSeq. The quality of sequencing raw materials was managed to be normalized for either mRNAs, Oligo-Abs, and sample tags, based on manufactory recommendations. Although according to the cells and expected amplitude of expression, both methods would theoretically be suitable for quantification of this assay, and both datasets were processed by the same platform and pipeline, a comparison of the outcome of two datasets from HiSeqX and NestSeq platforms showed that HiSeqX has a higher quality of cell detection, less imbalance population of cells between experimental conditions and more reads gained for proteomics detection. Both HiSeqX and NextSeq sequencing platforms were introduced by Illumina in early 2014 [24]. NextSeq system can produce a maximum of 400 million reads in a relatively short time, and HiSeq X, as a powerful sequencing system, has a much higher throughput of around 6 billion reads [24]. HiSeqX has an excessive yield of sequencing per run and costs more, which means that a larger amount of data (1800 Gb) can produce less raw error rate [25]. Both techniques have the same read length; they are both paired reads (2 × 150 bp are required for BD Rhapsody™ Single-Cell Analysis System) and the same insert size, which is capable both for high performances while single-cell sequencing conditions, of generating qualified genes, cell barcodes and unique molecular indexes [26]. Our observation was supported by previous cost-effective analysis suggesting that using HiSeq X sequencing technology generates a higher quality of base calls [27]. According to the two datasets analysis, we also can observe that NextSeq data have a certain number of qualified cells detected and can be used as downstream analysis with fewer data available; however, with deeper sequencing and more data generated, HiSeq X can produce a higher quality of data and detect about twice captured/qualified cells.

A significant cell number or percentage of lymphocyte subsets transforming due to the stimulation was observed while comparing experimental G1 and G2. Comparing the expressed mRNAs and surface proteins showed expected significantly differentially expressed variables between treated and untreated cell populations, especially for helper T cells and cytotoxic T cell populations. The transcriptomic patterns profile and surface proteins of helper and cytotoxic subsets have been dissected precisely in the current study by single-cell multi-omics after the anti-CD3 and CD28 stimulation (Figure 2) and were similar as reported before [21]. The important genes that represent cellular response for PCA components were also captured by our statistical analysis between G1 and G2, including IRF4, IFNG, and CD69 [21]. This notion has not been observed in some other single omics methods like CRISPR droplet single-cell RNA sequencing, which usually depicts lymphocyte subsets mixed with both unstimulated and stimulated cells and performs whole-transcriptome instead of targeted immune-transcriptome analysis [28]. Moreover, the advantage of our study on using Oligo-Abs for combining proteomics profiles is more efficient than defining cell types only by transcriptomics data. The merged multi-omics layer from the very same cell can help to present the states of that individual cell accurately.

Our current findings indicate that the basement stimulated and unstimulated control groups, G1 and G2, have the highest number of cells captured and qualified, compared with the rest groups, which were manipulated by fixation and permeabilization. Therefore, our finding supports the notion that not only does permeabilization have negative impacts on transcriptomics but also the fixation can decrease the detection of qualified cells. However, the cell population structure would be almost similar after fixation and permeabilization, and it does not make a bias toward a specific lymphocyte subset. The non-recapitulated significant expression parameters for the (G1–G2) pair in (G3–G4), (G5–G6), and (G7–G8) pairs comparisons have proved that the transcriptome data are more affected than proteomics losing because of the cellular damage by fixation and permeabilization before loading on the cartridge. RNA degradation and fragmentation may be caused by cell fixation of chemical modifications on nucleic acid structures and the opening of cell membranes [29,30]. Nuclease-free water has been used for following library preparation procedures after a single cell capture step, but still, fragile mRNAs retain on endoplasmic reticulum membrane-bound ribosomes they can be degraded by potential RNase in the environment, especially after the permeabilization step. Although fixation and permeabilization would have an adverse impact on the detection of the whole transcriptome for single-cell assay, both methods 1 and 2 detect about 60% of the signature of transcriptome correctly. Then, the trade-off needs to be managed by combining intracellular staining and transcriptome profiling. Moreover, these findings may suggest a trial of further adjustments in the timings of different permeabilization steps and a lower concentration of toxic agents for optimization of fixation and permeabilization.

Fixation and permeabilization method 2 detects the more precise proteomic fingerprint changes in the stimulation, compared with method 1. Fixation is often used to preserve samples for processing large numbers of samples to perform the same amount of next-generation sequencing or to prepare samples in which gene expression changes rapidly. Moreover, it can prevent autolysis and putrefaction and protect the tissue from damage during subsequent processing, which is crucial during intracellular staining and single-cell cartridge loading [31]. Most of the surface markers are detected in method 2, and a large amount of them are even significantly expressed due to the stimulation. Method 1, the BD company buffer, which is the theoretically optimized material for BD antibody staining and this single-cell system, was not performed as well as method 2. Method 2, fixed with 2% paraformaldehyde and permeabilized with Tween-20, reported by Amidzadeh et al. [23], was more efficient for maintaining the integrity of intracellular transcriptome after permeabilization. It also has been evident by the current experiment that method 2 can detect a higher amount of significant transcriptome fingerprints. However, the bias may arise because of the smaller number of cells captured and the low quality and filtration for experimental G6. The literature review on permeabilization methods on other single-cell technologies (mainly droplet-based methods) also showed similar challenges of both fixation and permeabilization. Optimal capturing methods for unperturbed state and long-term evolution of droplet-based single-cell RNA sequencing data were achieved by methanol fixation stabilizing and preserving embryologic and neuron cells with less cross-linking induction and lower inhibition of poly(dT) annealing [32]. However, another recent study by Van Phan et al. supported our current findings instead of the BD well-based method by fixed-droplet RNA sequencing of HEK293T cells detecting a higher number of genes and transcripts by paraformaldehyde than methanol fixation [33].

Despite the advantage of combining proteomics and transcriptomics, we must decrease the cell load on the BD Rhapsody platform. Therefore, the future perspective of the field will be on the expansion of cell numbers as new platforms can load up to a hundred thousand cells and more efficient sequencing platforms to cover both omics levels appropriately. Moreover, even though we showed that intracellular staining is impossible to merge with BD Rhapsody single-cell assay, due to the limitation of the current study and the availability of Oligo-Ab tagged for immune markers with an exclusive intracellular expression, we could not evaluate a simultaneous pattern of both intracellular and extracellular proteomics. So, in the future, it is clearer with a real experiment on the detection of specific short-term activation of downstream signaling to observe the state of the art of method 2 on the detection of intracellular proteomic signature. Few investigations tried to develop such intracellular Oligo-Abs and tested for single-cell RNA and Immunodetection (RIAD) [34] for phospho-protein detection (phosphorylated FAK levels) on keratinocytes only with 384-well plates. Although preliminary, they also suggested a low reduction in gene complexity and detectable biological differences between cell populations.

## 5. Conclusions

In conclusion, to combine surface and intracellular markers’ proteomics in immune cells, the best method for preserving the stimulation fingerprints for proteomics assays was fixation with 2% paraformaldehyde and permeabilization with Tween-20. This method is applicable but needs to be optimized further in future studies to balance detecting fewer cells and transcriptome loss with intracellular antibody staining. However, the development of Oligo-Abs for immune intracellular targets is the current barrier, which should be produced soon by specialized core facilities.

## Figures and Tables

**Table 1:** Eight different conditions designed to assess the influence of fixation and permeabilization on the BD Rhapsody™ Single-Cell Analysis System. The number of captured cells and qualified cells detected within each experimental group are depicted. All conditions were generated at one experiment without replicates using multiplexing barcoding to minimize antibody staining errors, and sequencing was performed from the same molecule pool in two different platforms of Nextseq and HiseqX (generating technical replicates). The differences observed in the final captured/qualified cells depend on each experimental design despite equal loading cell proportions.

ID	Group	Experimental Conditions	Captured Cells		Qualified Cells		% of Filtration	
			HiSeq	NextSeq	HiSeq	NextSeq	HiSeq	NextSeq
Experimental condition 1	G1	Unstimulated group	128	24	113	18	11.7	25
Experimental conditions 2	G2	Stimulated group	193	87	183	84	5.2	3.4
Experimental condition 3	G3	Unstimulated + Fixation group	59	35	54	29	8.5	17.1
Experimental condition 4	G4	Stimulated + Fixation group	82	49	78	48	4.9	2
Experimental condition 5	G5	Unstimulated + Fixation/Permeabilization method 1 group	91	68	87	61	4.4	10.3
Experimental condition 6	G6	Stimulated + Fixation/Permeabilization method 1 group	39	20	35	19	10.3	5
Experimental condition 7	G7	Unstimulated + Fixation/Permeabilization method 2 group	79	66	77	54	2.5	18.2
Experimental condition 8	G8	Stimulated + Fixation/Permeabilization method 2 group	80	70	75	61	6.2	10

**Table 2:** Two sequencing datasets comparison between Nextseq and HiSeqX platforms.

Parameters	HiSeqX	NextSeq
Number of loaded cells (technical replicates)	1000 ^a^	
Number of captured cells	751	419
Number of total reads in FASTQ	273,868,263	112,868,918
Number of total reads in total filtered reads	268,858,136	112,353,767
Number of total mRNAs reads	1,083,132	283,449
Number of total Oligo-Abs reads	194,121,222	82,018,987
Number of unique mRNAs detected	6893	2899
Number of qualified cells	702	374
% of filtration	6.5	10.7
Library size median (IQR)	53 (32–88)	34 (27–51)
Gene expressed median (IQR)	35 (24–56)	26 (22–37)
Number of mRNA left from 2.5% to 97.5% of cell expressed density	2459	1053
Number of mRNA used for clustering	62	27

^a^ Based on the explained method, we aligned and pooled every 8 conditions with final cell counts of 8000 cells (1000 cells/condition); there were 1000 final loaded cells on the single-cell cartridge.

**Table 3:** Median and IQR values for 15 detected Oligo-Abs (pAO).

Antibodies	Median		Interquartile Range		0.25 Quantile		0.75 Quantile	
	HiSeq	NextSeq	HiSeq	NextSeq	HiSeq	NextSeq	HiSeq	NextSeq
CD45RA.pAO	189	148	496	268.5	67.5	55.5	563.5	324
CD4.pAO	307	167	720	291	111	83	831	374
CD38.pAO	388	245	505.5	270	211.5	138.5	717	408.5
CD56.pAO	261	259	552.5	321	53.0	119.5	605.5	440.5
CD19.pAO	243	214	582	262.5	18	138	600	400.5
IgD.pAO	304	302	634.5	303	60.5	174	695	477
CD27.pAO	552	348	998	464	241.5	202.5	1239.5	666.5
CD25.pAO	587	376	985	548.5	235.5	234	1220.5	782.5
CD3.pAO	639	452	1219	668	253.5	235	1472.5	903
CD8.pAO	788	593	1698.5	823.5	157	352.5	1855.5	1176
CD16.pAO	1107	905	2352.5	963.5	234	567.5	2586.5	1531
CD45.pAO	3557	1554	3902.5	1801.5	1843	686.5	5745.5	2488
CD197.pAO	1704	1447	3677.5	1841	174	1051	3851.5	2892
CD127.pAO	**948**	**2346**	**5797.5**	**4029**	**131.5**	**544.5**	**5929**	**4573.5**
TCR-γδ.pAO	4482	4191	10,269.5	5578	539.0	2642.5	10,808.5	8220.5

Bold depict the marker with higher median of detected protein in the Nextseq compared to HiSeq.

## Data Availability

The datasets presented in this study can be found in online Supplementary Data. The link to the repository/and accession number(s) can be found below: https://www.ebi.ac.uk/ena/browser/view/PRJEB46204?show=reads (accessed on 23 July 2024).

## Supplementary Materials

The following are available online at https://www.mdpi.com/article/10.3390/antib14010015/s1. Table S1: Eight different Sample Tags used with common 5′ and 3′ ends in the study, Table S2: Oligo-Ab used for assessing the influence of fixation and permeabilization on the BD Rhapsody™ Single-Cell Analysis System, Table S3: Sequencing flow cell loading and PhiX concentrations, Table S4: Comparison of observed vs. expected qualified captured cells between experimental conditions using a chi-square test goodness of fit (*p*-values are presented), Table S5: The mRNA with the highest dispersion selected for the variable reduction, Table S6: Surface markers used for supervised classification of cell types, Table S7: The *p*-values of the chi-square test which comparing each two group’s cell numbers for four cell types of differences, Table S8: The significant parameters detected while comparing group 1 and group 2 within four main lymphocyte/cell-type populations, Table S9: The significant parameters detected while comparing group 1 and group 3 within four cell-type populations, Table S10: The significant parameters detected while comparing group 1 and group 5 within four cell-type populations, Table S11: The significant parameters detected while comparing group 1 and group 7 within four cell-type populations, Table S12: The significant parameters detected while comparing group 3 and group 4 within four cell-type populations, Table S13: The significant parameters detected while comparing group 5 and group 6 within four cell-type populations, Table S14: The significant parameters detected while comparing group 7 and group 8 within four cell-type populations, Table S15: the significant expressed parameters in group 1 and group 2 comparison for helper T cells, Table S16: the significant expressed parameters in group 1 and group 2 comparison for cytotoxic T cells, Table S17: the significant expressed parameters in group 1 and group 2 comparison for B cells, Table S18: the significant expressed parameters in group 1 and group 2 comparison for NK/T cells, Table S19: Comparative approach considering condition G1 vs. G2 as gold standard and define differentially expressed markers for each cell type subset indicating the fraction of recovered differentially expressed genes in experimental conditions (G3 vs. G4, G5 vs. G6, and G7 vs. G8) comparison with the gold standard, Figure S1: Quality controls for single-cell multi-omics data generated on selected permeabilization/fixation methods on lymphocytes, Figure S2: Visualizations of the top significant parameters in each cell type while comparing group 1 and group 2, Figure S3: Volcano plots of comparing differential expression of cluster 1 vs. cluster 2 (C1 vs. C2), C1 vs. C3, C1 vs. C4, Figure S4: T-SNE plot of experimental conditions 1 and 3 based on the transformed expression of the 6 lineage markers in the cells from the PBMC dataset to detect the impact of fixation, Figure S5: T-SNE plot of experimental conditions 1 and 5 based on the transformed expression of the 6 lineage markers in the cells from the PBMC dataset to detect the impact of permeabilization method 1 and fixation, Figure S6: T-SNE plot of experimental conditions 1 and 7 based on the transformed expression of the 6 lineage markers in the cells from the PBMC dataset to detect the impact of permeabilization method 2 and fixation, Figure S7: Visualizations of the top significant parameters in each cell type while comparing group 1 and group 3, Figure S8: Visualizations of the top significant parameters in each cell type while comparing group 1 and group 5, Figure S9: Visualizations of the top significant parameters in each cell type while comparing group 1 and group 7, Figure S10: T-SNE plot of experimental conditions 3 and 4 based on the transformed expression of the 6 lineage markers in the cells from the PBMC dataset to detect the impact of stimulation, Figure S11: Visualizations of the top significant parameters in each cell type while comparing group 3 and group 4, Figure S12: T-SNE plot of experimental conditions 5 and 6 based on the transformed expression of the 6 lineage markers in the cells from the PBMC dataset to detect the impact of stimulation, Figure S13: Visualizations of the top significant parameters in each cell type while comparing group 5 and group 6, Figure S14: T-SNE plot of experimental conditions 7 and 8 based on the transformed expression of the 6 lineage markers in the cells from the PBMC dataset to detect the impact of stimulation, Figure S15: Visualizations of the top significant parameters in each cell type while comparing group 7 and group 8.
