# Supplementary material for: Assessing the Influence of Selected Permeabilization Methods on Lymphocyte Single-Cell Multi-Omics
Source: Antibodies (Basel). 2025 Feb 10;14(1):15. doi: 10.3390/antib14010015 (PMC11843891; doi:10.3390/antib14010015)

Supplementary Materials

Assessing the Influence of Selected Permeabilization Methods on Lymphocyte Single-Cell Multi-Omics

Supplementary Methods.

Detailed bioinformatic analysis have been performed on the output file of NextSeq and HiseqX sequencing platforms. After gaining the Fastq file (both-side reads R1 and R2) as sequencing result from Illumina sequencer, the raw data was analyzed by the Seven Bridges Genomics platform (BD Rhapsody™ WTA Analysis Pipeline). Quality control was done by using this pipeline, including read-quality filtering, validating read pairs and collapsing reads to raw molecules. The Unique Molecular Identifier (UMI) has been designed to label different mRNA and Oligo-Ab attached to a specific bead. Therefore, UMI errors were adjusted by recursive substitution error correction (RSEC) which may be caused by single-base substitution errors. Then, distribution-based error correction (DBEC) was performed to correct library preparation errors or sequencing base deletions. In the next step, the bead label, which was used for labeling each cell with a different expression profile was detected. Thus, UMIs were assigned to their original cells, and the cells, which did not have an expression profile were filtered out. After identifying the cell labels, the sample tags which labeled eight experimental conditions were used to classify cells belonging to each condition. Subsequently, the single-cell gene expression profiles were aligned (GRCh38-PhiX-gencodev29.tar.gz and AbSeq_AbOligoReference.fasta) and annotated (Gencodev29-20181205.gtf) using Bowtie2 to generate SAM file as an output file. The SAM file was converted to the BAM file, and the metrics of the analysis, including the counts of mRNA and Oligo-Ab for every single cell, were reported.

The cells, which were assigned to certain experimental sample tags, remained as input matrix, excluding the cells that had undetermined sample tags or multiple sample tags. For unsupervised clustering analysis, quality control was implemented to filter out outlier cells based on their mRNA expression of targeted genes (immune targets transcriptome analysis). The outlier cells were the cells, that were not in the range of 2.5% to 97.5% of the log-transformed normalized distribution of library size (the sum of the expressed mRNAs molecules for each cell) and gene expression (total number of unique mRNA expressed within each cell). Next, to achieve a better clustering of the cells, variable reduction for selecting the representative mRNAs was performed according to 2.5% to 97.5% log-transformed normalized distribution of cell-expressed (total number of unique cells expressing the specific mRNAs) and then selecting the top 2.5% mRNAs with the highest dispersion (variance/mean, to identify the highly differentially expressed genes). By using the selected mRNAs, combined with

the captured Oligo-Abs, principal component analysis (PCA) and t-distributed stochastic neighbor embedding (t-SNE) were performed for dimensionality reduction and visualization. Then manually added polygons in the t-SNE space were used for defining the main cell clusters. Supervised classification was achieved by defining the main cell/immune markers in each identified unsupervised cluster.

Table S1. Eight different Sample Tags used with common 5' and 3' ends in the study.

**Sample Tag**	**Sample Tag sequence**
Sample Tag 1— Human	ATTCAAGGGCAGCCGCGTCACGATTGGATACGACTGTTGGACCGG
Sample Tag 2— Human	TGGATGGGATAAGTGCGTGATGGACCGAAGGGACCTCGTGGCCGG
Sample Tag 3— Human	CGGCTCGTGCTGCGTCGTCTCAAGTCCAGAAACTCCGTGTATCCT
Sample Tag 4— Human	ATTGGGAGGCTTTCGTACCGCTGCCGCCACCAGGTGATACCCGCT
Sample Tag 5— Human	CTCCCTGGTGTTCAATACCCGATGTGGTGGGCAGAATGTGGCTGG
Sample Tag 6— Human	TTACCCGCAGGAAGACGTATACCCCTCGTGCCAGGCGACCAATGC
Sample Tag 7— Human	TGTCTACGTCGGACCGCAAGAAGTGAGTCAGAGGCTGCACGCTGT
Sample Tag 8— Human	CCCCACCAGGTTGCTTTGTCGGACGAGCCCGCACAGCGCTAGGAT

Table S2- Oligo-Ab used for assessing the influence of fixation and permeabilization on the BD Rhapsody™ Single-Cell Analysis System.

**Antibodies**	**Clones**	**Antibodies**	**Clones**
Oilgo-Mouse Anti-Human CD3	SK7 (also known as Leu-4) Size:25 Tests Status (RUO)	Oilgo-Mouse Anti-Human CD38	HIT2 Size:25 Tests Status (RUO)
Oilgo-Mouse Anti-Human CD4	RPA-T4 (RUO)	Oilgo-Mouse Anti-Human CD197	2-L1-A (RUO)
Oilgo-Mouse Anti-Human CD45	HI30 (RUO)	Oilgo-Mouse Anti-Human CD27	M-T271 Size:25 Tests Status (RUO)
Oilgo-Mouse Anti-Human CD8	RPA-T8	Oilgo-Mouse Anti-Human CD45RO	UCHL1 (RUO)
Oilgo-Mouse Anti-Human CD19	SJ25C1 (also known as SJ25-C1) (RUO)	Oilgo-Mouse Anti-Human IgD	IA6-2 (also known as δ-IA6-2) (RUO)
Oilgo-Mouse Anti-Human CD16	3G8 Size:25 Tests Status (RUO)	Oilgo-Mouse Anti-Human CD21	B-ly4 Size:25 Tests Status (RUO)
Oilgo-Mouse Anti-Human CD56	NCAM16.2 (also known as NCAM 16) Size:25 Tests Status (RUO)	Oilgo-Mouse Anti-Human TCR-αβ	IP26 (also known as IP26A) (RUO)
Oilgo-Mouse Anti-Human CD25	2A3 Size:25 Tests Status (RUO)	Oilgo-Mouse Anti-Human TCR-γδ	B1 (RUO)
Oilgo-Mouse Anti-Human CD45RA	HI100 Size:25 Tests Status (RUO)	Oilgo-Mouse Anti-Human CD314	1D11 (RUO)
Oilgo-Mouse Anti-Human CD127	HIL-7R-M21 (RUO)	Oilgo-Mouse Anti-Human CD94	HP-3D9 (RUO)

Table S3- Sequencing flowcell loading and PhiX concentrations

Illumina system	Sequencing flowcell loading concentration	PhiX concentration
NextSeq High Output	1–1.5 pM	20%
HiSeq-X	3 nM	15%

Table S4– Comparison of observed vs. expected qualified captured cells between experimental conditions using a chi-square test goodness of fit (*p*-values are presented).

Experiment ID	G1	G2	G3	G4	G5	G6	G7
**G1**							
**G2**	4.728e-05						
**G3**	4.982e-06	2.2e-16					
**G4**	0.01132	8.067e-11	0.03671				
**G5**	0.06599	5.146e-09	0.005451	0.4835			
**G6**	1.44e-10	2.2e-16	0.04401	5.23e-05	2.503e-06		
**G7**	0.009009	4.903e-11	0.04448	0.936	0.4349	7.229e-05	
**G8**	0.005581	1.771e-11	0.06447	0.8084	0.3458	0.0001368	0.8711

Table S5 – The mRNA with the highest dispersion selected for the variable reduction.

Genes						
AAGAB	*AQP7*	*CAMK1G*	*CLEC2D*	*EBNA1BP2*	*IGKC*	*MAGEA8*
AC005261.1	*ARHGAP9*	*CCL2*	*CLK4*	*EIF3E*	*IGLC2*	*MAP3K13*
AC020917.3	*ARL6IP1*	*CCL22*	*CMTM2*	*FAU*	*IL1B*	
AC079035.1	*ARPC2*	*CCL3*	*CUL9*	*FCGRT*	*IL9*	
AC103736.1	*ASF1B*	*CCL4*	*CXCL10*	*GLB1*	*ILF2*	
ADRA1A	*ATP5MGL*	*CCL5*	*CXCL8*	*GRK6*	*IPO4*	
AFMID	*C12orf76*	*CD47*	*CXCL9*	*HLA.E*	*ITGAM*	
AGPAT3	*C2orf42*	*CENPM*	*DDA1*	*HMOX2*	*KCNE4*	

AL589678.1	*CA12*	*CENPT*	*DNAJC2*	*HSBP1*	*KLHL41*	
AP003025.1	*CALR*	*CHCHD2*	*DYNC1H1*	*IFNG*	*LARP4B*	

Table S6– Surface markers used for supervised classification of cell types.

**Cell type classification**	**immunologic markers**
Helper T cells	$CD3^{+}CD4^{+}$
Cytotoxic T cells	$CD3^{+}CD8^{+}$
B cells	$CD3^{-}$ $CD19^{+}$
NK/T cells	$CD3^{+/-}$, $CD4^{+/-}$, $CD16^{+}/CD56^{+}$

Table S7 – The *p*-values of the chi-square test which comparing each two group's cell numbers for four cell types of differences.

**Cell types**	**G1 vs G2**	**G1 vs G3**	**G1 vs G5**	**G1 vs G7**	**G3 vs G4**	**G5 vs G6**	**G7 vs G8**
B	0.13	1	0.84	1	1	1	1
NK/T	0.33	0.32	0.35	0.56	0.98	1	0.47
Helper T	0.68	0.63	0.75	0.7	1	1	1
Cytotoxic T	0.58	1	1	1	1	1	1

Table S8. – The significant parameters detected while comparing group 1 and group 2 within four main lymphocyte/cell-type populations.

B cells				NK/T cells				Helper T cells				Cytotoxic T cells			
Parameters	G1_mean	G2_mean	*p*_values	Parameters	G1_mean	G2_mean	*p*_values	Parameters	G1_mean	G2_mean	*p*_values	Parameters	G1_mean	G2_mean	*p*_values
CD25. pAbO	469.3	1492.1	0.00014	FCN1	0.38	0	0.0022	CD4.pAbO	617.96	2912.55	3.70E-16	CD27. pAbO	636.39	4588.41	1.30E-10
CD27. pAbO	538.6	3089.3	0.00036	IFNGR1	0.33	0	0.0049	CD27.pAbO	417.58	5111.25	9.30E-13	CD8. pAbO	1973.25	5759.18	1.10E-09
CD45RA. pAbO	251	539.0	0.0066	ITGAX	0.33	0	0.0049	CD25. pAbO	334.75	2426.77	7.10E-10	CD45RA. pAbO	354.75	1059.82	3.20E-07
IFNGR1	0.19	0	0.022	CCL2	0.38	0	0.017	CD45RA. pAbO	160.96	527.66	1.00E-05	CD8A	0	0.79	2.30E-06
AC003681.1	0.15	0	0.043	LILRB4	0.29	0	0.03	CD16. pAbO	3135.88	173.39	3.50E-05	CD25. pAbO	516.71	1822.56	3.60E-05
ADA	0	0.14	0.043	LY86	0.33	0	0.031	LCK	0	0.52	0.00012	CD4. pAbO	291.64	79.33	0.00014
CNTLN	0.15	0	0.043	FCGR3A	0.52	0.12	0.037	LEF1	0	0.41	0.00032	LEF1	0	0.33	0.00089
ICOS	0	0.14	0.043	CD14	0.19	0	0.042	DMXL1	0	0.27	0.0018	CD16. pAbO	2277.57	605.95	0.003
LY86	0.15	0	0.043	CNTLN	0.19	0	0.042	DUSP4	0	0.23	0.0028	C20orf96	0	0.21	0.0033
MIXL1	0.15	0	0.043					IRF4	0	0.34	0.0056	CD27	0	0.31	0.0033
								LAT	0	0.23	0.0061	CD8B	0.11	0.87	0.004
								CXCR3	0	0.16	0.0066	CD56.p AbO	785.25	210.82	0.0042
								CD3. pAbO	452.54	855.73	0.0078	CTSW	0	0.18	0.0064
								LY86	0.38	0	0.0093	DUSP2	0	0.33	0.0075

		BIRC3	0	0.18	0.0097	CD6	0	0.33	0.01
		CD247	0	0.18	0.0097	CXCR3	0	0.23	0.011
		CD6	0	0.23	0.011	MCM4	0	0.23	0.011
		IFITM3	0	0.23	0.011	CD127. pAbO	2637	741.44	0.011
		IFNGR1	0.29	0.02	0.011	TCRgd. pAbO	9662.68	2850.67	0.011
		CD56. pAbO	571.29	53.32	0.011	CD197. pAbO	3268.39	972.49	0.013
		GDF5OS	0	0.14	0.013	LAT	0	0.28	0.014
		CD127. pAbO	2113.79	162.89	0.013	CD44	0	0.18	0.018
		CD8. pAbO	947.92	127.43	0.015	IFITM3	0	0.18	0.018
		ICOS	0	0.23	0.017	IRF4	0.04	0.31	0.018
		MAP4K4	0	0.16	0.018	CSF2	0	0.38	0.02
		TCRgd.pAbO	6877.79	560.39	0.018	LCK	0.04	0.28	0.021
		CD197. pAbO	2506.62	211.07	0.019	KCNJ3	0	0.13	0.023
		CD69	0	0.11	0.024	CD45.pAbO	4428.07	5794.87	0.029
		IL4R	0	0.2	0.027	BIRC3	0.07	0.36	0.03
		IFITM2	0.04	0.27	0.029	CBLB	0	0.15	0.032
		AL136454.1	0	0.18	0.031	GDF5OS	0	0.15	0.032

		CD9	0.25	0	0.031	IFITM2	0.04	0.26	0.035
		CD27	0	0.14	0.032	CATSPER2	0.14	0	0.043
		DUSP2	0.04	0.2	0.032	CNTLN	0.14	0	0.043
		FCGR3A	0.29	0	0.032	MIXL1	0.14	0	0.043
		FCN1	0.42	0	0.038	AL136454.1	0	0.31	0.044
		CNTLN	0.17	0	0.043	CD200	0	0.1	0.044
		FTH1	0.33	0.02	0.043	CD37	0	0.1	0.044
		APOBEC3G	0	0.09	0.044	DUSP4	0	0.1	0.044
		IKZF1	0	0.09	0.044	MAP4K4	0	0.1	0.044
		IL12RB2	0	0.09	0.044	LIF	0	0.23	0.048

Table S9– The significant parameters detected while comparing group 1 and group 3 within four cell-type populations.

B cells				NK/T cells				Helper T cells				Cytotoxic T cells			
Parameters	G1_mean	G3_mean	*p*_values	Parameters	G1_mean	G3_mean	*p*_values	Parameters	G1_mean	G3_mean	*p*_values	Parameters	G1_mean	G3_mean	*p*_values
CD127. pAbO	2810.3	8269.0	5.0E-06	FCN1	0.38	0	0.0022	CD3.pAbO	452.5	1275.4	0.00061	CD127. pAbO	2637	6103.2	0.019
IFNGR1	0.19	0	0.022	FCGR3A	0.52	0	0.0045	CD45.pAbO	7092.1	4000.5	0.00088	CNTLN	0.14	0	0.043
CD25. pAbO	469.3	687.6	0.036	IFNGR1	0.33	0	0.0049	CD4.pAbO	617.96	1090.2	0.0021				
CD19. pAbO	453.6	682.5	0.042	ITGAX	0.33	0	0.0049	IFNGR1	0.29	0	0.0053				
CATSPER2	0.15	0	0.043	CCL2	0.38	0	0.017	LY86	0.38	0	0.0093				
CYTLN	0.15	0	0.043	LILRB4	0.29	0	0.03	CD16. pAbO	3135.8	1553.4	0.017				
LY86	0.15	0	0.043	LY86	0.33	0	0.031	FTH1	0.33	0	0.029				
				CD14	0.19	0	0.042	CD9	0.25	0	0.031				
				CDC14B	0.19	0	0.042	CXCL8	0.29	0	0.032				
				CNTLN	0.19	0	0.042	FCGR3A	0.29	0	0.032				
								FCN1	0.42	0	0.038				
								CDC14B	0.17	0	0.043				
								CNTLN	0.17	0	0.043				

Table S10 – The significant parameters detected while comparing group 1 and group 5 within four cell-type populations.

B cells				NK/T cells				Helper T cells				Cytotoxic T cells			
**Parameters**	**G1_mean**	**G5_mean**	***p*_values**	**Parameters**	**G1_mean**	**G5_mean**	***p*_values**	**Parameters**	**G1_mean**	**G5_mean**	***p*_values**	**Parameters**	**G1_mean**	**G5_mean**	***p*_values**
CD3.pAbO	549.85	1908.95	5.90E-07	FCN1	0.38	0	0.0022	CD3.pAbO	452.54	2001.62	2.00E-10	CD3.pAbO	618.39	1769.05	7.70E-07
CD45RA.pAbO	251	796.32	5.80E-05	FCGR3A	0.52	0	0.0045	CD4.pAbO	617.96	1405.95	6.80E-09	CD45RA.pAbO	354.75	844.33	0.00032
CD4.pAbO	323.04	854.77	0.00011	IFNGR1	0.33	0	0.0049	CD4SRA.pAbO	160.96	555.52	810E-05	CD8.pAbO	1973.25	3184	0.011
CD27.pAbO	538.65	1089.36	0.00028	ITGAX	0.33	0	0.0049	CD27.pAbO	417.58	942.86	0.00085	CD27.pAbO	636.39	993	0.023
CD25.pAbO	469.38	1094.59	0.00081	CCL2	0.38	0	0.017	CD45.pAbO	7092.12	4637.14	0.003	CD4.pAbO	291.64	519.14	0.029
lgD.pAbO	666.27	1278.41	0.0047	LILRB4	0.29	0	0.03	CD25.pAbO	334.75	758.38	0.0035	CD25.pAbO	516.71	950.67	0.037
CD19.pAbO	453.65	798.23	0.016	LY86	0.33	0	0.031	IFNGR1	0.29	0	0.0053	ACAP2	0	0.19	0.042
ALS2CR12	0	0.23	0.021	LAMB1	0	0.36	0.038	LY86	0.38	0	0.0093	FAAP24	0	0.19	0.042
ANAPC1	0	0.23	0.021	CD14	0.19	o	0.042	ACSL6	0	0.24	0.021	FBXL17	0	0.19	0.042
IFNGR1	0.19	0	0.022	CXCL8	0.19	0	0.042	lgD.pAbO	477.92	965.67	0.025	MIXL1	0.14	0	0.043
CD8.pAbO	1475.54	2424.41	0.03					FTH1	0.33	0	0.029				

ACAP2	0	0.18	0.042		CD9	0.25	0	0.031	
AP002360.2	0	0.18	0.042		CXCL8	0.29	0	0.032	
FBXL17	0	0.18	0.042		FCGR3A	0.29	0	0.032	
GLCCI1	0	0.18	0.042		FCN1	0.42	0	0.038	
LAMB1	o	0.18	0.042		AC127024.2	0	0.19	0.042	
AC003681.1	0.15	0	0.043		ADAMTS9.AS2	0	0.19	0.042	
LY86	0.15	0	0.043						
MIXL1	0.15	0	0.043						

Table S11– The significant parameters detected while comparing group 1 and group 7 within four cell-type populations.

B cells				NK/T cells				Helper T cells				Cytotoxic T cells			
**Parameters**	**G1_mean**	**G7_mean**	***p*_values**	**Parameters**	**G1_mean**	**G7_mean**	***p*_values**	**Parameters**	**G1_mean**	**G7_mean**	***p*_values**	**Parameters**	**G1_mean**	**G7_mean**	***p*_values**
CD127.pAbO	2810.38	15638.17	3.80E-11	TCRgd.pAbO	241.5	11460	0.00036	CD3.pAbO	452.54	2279.79	1.80E-10	CD127.pAbO	2637	13291.89	2.00E-06
CD19.pAbO	453.65	1126.39	2.80E-05	FCN1	0.38	0	0.0022	CD45.pAbO	7092.12	1343.84	2.40E-10	CD45.pAbO	4428.07	2042.37	1.00E-04
CD45.pAbO	5031.65	1970.44	0.00022	FCGR3A	0.52	0	0.0045	CD127.pAbO	2113.79	10263.53	9.50E-06	CD3.pAbO	618.39	1713.16	3.00E-04
CD197.pAbO	3500.5	6802.28	0.0016	IFNGR1	0.33	0	0.0049	CD4.pAbO	617.96	917.16	0.00018	FST	0.04	0.37	0.011
CD25.pAbO	469.38	790.06	0.0065	ITGAX	0.33	0	0.0049	IFNGR1	0.29	0	0.0053	CD197.pAbO	3268.39	5967.26	0.014
FST	0.04	0.39	0.01	CCL2	0.38	0	0.017	LY86	0.38	0	0.0093	TCRgd.pAbO	9662.68	16810.63	0.025
IFNGR1	0.19	0	0.022	LILRB4	0.29	0	0.03	FTH1	0.33	0	0.029	GABPB1.AS1	0	0.21	0.042
LY86	0.15	0	0.043	LY86	0.33	0	0.031	CD9	0.25	0	0.031				
				CD14	0.19	0	0.042	CKCL8	0.29	0	0.032				
				CXCL8	0.19	0	0.042	FCGR3A	0.29	0	0.032				
								FCN1	0.42	0	0.038				
								CDC14B	0.17	0	0.043				

Table S12 – The significant parameters detected while comparing group 3 and group 4 within four cell-type populations.

B cells				NK/T cells				Helper T cells				Cytotoxic T cells			
**Parameters**	**G3_mean**	**G4_mean**	***p*_values**	**Parameters**	**G3_mean**	**G4_mean**	***p*_values**	**Parameters**	**G3_mean**	**G3_mean**	***p*_values**	**Parameters**	**G3_mean**	**G4_mean**	***p*_values**
CD19.pAbO	682.54	1268.1	1.90E-08	CD16.pAbO	2232.17	5187.5	0.00095	CD197.pAbO	2618.57	7279.79	9.80E-06	CD25.pAbO	607.79	2891.42	1.20E-07
CD197.pAbO	4914.46	9553.6	2.00E-08	CD197.pAbO	3990.83	8885	0.0029	TCRgd.pAbO	7546.71	22657.95	1.00E-05	CD3.pAbO	867.79	1894.32	7.40E-06
lgD.pAbO	728.15	1280.75	2.60E-07	CD25.pAbO	606.17	3675.2	0.0032	CD4.pAbO	1090.29	2081.74	2.20E-05	CD8.pAbO	2116.71	3877.05	4.60E-05
CD127.pAbO	8269.08	15219.2	3.70E-06	CD56.pAbO	604	916.3	0.0048	CD25.pAbO	436.71	2952.84	2.50E-05	CD27.pAbO	755.71	1408.05	0.0013
CD25.pAbO	687.62	3195.15	6.80E-06	CD27.pAbO	748.33	1607.6	00073	CD27.pAbO	546.14	1367.05	3.70E-05	TCRgd.pAbO	11368.21	22999.53	0.0038
CD27.pAbO	723.69	1546.35	6.80E-06	CD45.pAbO	4262.17	7110.8	0.0088	CD8.pAbO	716.86	1621.05	7.40E-05	CD16.pAbO	2077.57	3836.95	0.0051
CD38.pAbO	478.54	825.85	0.00059	CD127.pAbO	5903.67	13636.4	0.01	CD127.pAbO	3467.43	10690	0.00011	CD197.pAbO	3934.64	7345.11	0.0055
ERBB3	o	0.3	0.01	CD45RA.pAbO	233.17	559.4	0.026	CD16.pAbO	1553.43	3613.74	0.00013	CD56.pAbO	553.64	983.89	0.021
AC097376.2	o	0.25	0.021	FAM135A	0	0.4	0.037	CD56.pAbO	439	741.47	0.00078	CNTLN	0	0.32	0.03
HOTTIP	o	0.25	0.021	GPM6B	0	0.4	0.037	CD38.pAbO	484.86	793.26	0.0044	CD127.pAbO	6103.29	10744.89	0.036

FBXO33	o	0.3	0.03		CD45RA.pAbO	273.93	544.16	0.0054	CD45RA.pAbO	416.21	709.84	0.039
GPM6B	0	0.3	0.03		CD45.pAbO	4000.57	6149.63	0.0059	MIRLET7BHG	0.29	o	0.04
IREB2	0	0.3	0.03		CEP41	0	0.26	0.021	CCDC58	o	0.21	0.042
ADD3	o	0.2	0.042		CNTLN	o	0.26	0.021	DIS3L	o	0.21	0.042
AMMECR1	o	0.2	0.042		CAST	0	0.32	0.03	IFI44L	0	0.21	0.042
CEP41	0	0.2	0.042		ACAP2	0	0.21	0.042				
DIS3L	0	0.2	0.042		DCAF10	0	0.21	0.042				
FADS3	0	0.2	0.042									
FAM135A	0	0.2	0.042									
FST	o	0.2	0.042									
ME2	o	0.2	0.042									

Table S13 – The significant parameters detected while comparing group 5 and group 6 within four cell-type populations.

B cells				NK/T cells				Helper T cells				Cytotoxic T cells			
**Parameters**	**G5_mean**	**G6_mean**	***p*_values**	**Parameters**	**G5_mean**	**G6_mean**	***p*_values**	**Parameters**	**G5_mean**	**G6_mean**	***p*_values**	**Parameters**	**G5_mean**	**G6_mean**	***p*_values**
ALS2CR12	0.23	0	0.021	LAMB1	0.36	0	0.038	ADAMTS9	0.19	0	0.042	DPP6	0.19	0	0.042
ANAPC1	0.23	0	0.021									LINC01411	0.19	0	0.042
ARHD18	0.27	0	0.03												
ADAMTS9	0.18	0	0.042												
CDK13	0.18	0	0.042												
DPP6	0.18	0	0.042												
ELAVL3	0.18	0	0.042												
FBXL17	0.18	0	0.042												
FBXO33	0.18	0	0.042												
GLCCI1	0.18	0	0.042												
LAMB1	0.18	0	0.042												

Table S14 – The significant parameters detected while comparing group 7 and group 8 within four cell-type populations.

**B cells**				**NK/T cells**				**Helper T cells**				**Cytotoxic T cells**			
**Parameters**	**G7_mean**	**G8_mean**	***p*_values**	**Parameters**	**G7_mean**	**G8_mean**	***p*_values**	**Parameters**	**G7_mean**	**G8_mean**	***p*_values**	**Parameters**	**G7_mean**	**G8_mean**	***p*_values**
CD25.pAbO	790.06	1527.94	2.40E-08	CD25.pAbO	665.45	1516.8	8.20E-06	CD25.pAbO	507.26	1321.22	1.20E-05	CD25.pAbO	671.16	1504.84	1.90E-07
CD127.pAbO	15638.17	28061.71	4.10E-07	CD27.pAbO	603.73	1236.87	0.00026	CD16.pAbO	2150.74	4685.61	0.00016	CD3.pAbO	1713.16	3161.89	2.40E-05
CD197.pAbO	6802.28	11708.35	810E-07	CD16.pAbO	2891.27	5517.27	0.00059	CD8.pAbO	933.84	2217.5	0.00022	CD197.pAbO	5967.26	10736.21	7.90E-05
CD19.pAbO	1126.39	1926.88	1.20E-06	CD56.pAbO	660.73	1767.73	0.0012	CD27.pAbO	469.42	1072.06	0.00033	CD16.pAbO	2934.42	5331.05	8.70E-05
IgD.pAbO	927.39	1385.06	0.00051	CD197.pAbO	5881.09	10479.13	0.0019	CD197.pAbO	4671.84	9123.56	0.00059	TCRgd.pAbO	16810.63	29962.37	0.00034
CD27.pAbO	777.44	1064.29	0.0065	TCRgd.pAbO	16487.91	29629.47	0.0029	CD56.pAbO	462.58	1517.33	0.00084	CD56.pAbO	658	1538.79	0.0018
CD82	0	0.29	0.02	CD3.pAbO	1380.55	2713.53	0.004	TCRgd.pAbO	12879.63	25548	0.0011	CD27.pAbO	742.53	1167.26	0.0031
ATG12	0	0.24	0.041	CD127.pAbO	12731.18	23041.2	0.007	CD4.pAbO	917.16	683.33	0.0012	CD127.pAbO	13291.89	22890.63	0.0039
CALD1	0	0.24	0.041	CD38.pAbO	567.27	1469	0.008	CD38.pAbO	422.53	1271.56	0.0049	CD82	o	0.37	0.0045

LINCO1096	0	0.24	0.041	CD45RA.pAb O	131.91	360.13	0.012	CD127.pAbO	10263.53	19242.22	0.0071	CD38.pAbO	586.68	1279.79	0.012
DUSP18	0.11	0.41	0.047	CAND1	0	0.33	0.019	CD45RA.pAb O	109.53	316.83	0.0078	CYP3A5	0	0.37	0.031
				LHG1	0	0.33	0.019	CD45.pAbO	1343.84	2316.44	0.01	AC097376.2	0.05	0.42	0.039
				CYP3AS	0	0.47	0.029	CAND1	0	0.28	0.02	DUSP18	0.05	0.32	0.04
				AC091614.1	0	0.27	0.041	LHG1	0	0.28	0.02	AC091614.1	0	0.21	0.042
								CD3.pAbO	2279.79	2896.94	0.02	CEP41	0.21	0	0.042
								CYP3AS	0	0.39	0.03	EIF4E	0	0.21	0.042
								CADPS	0	0.22	0.042	MAP2K6	o	0.21	0.042
								CD82	0	0.22	0.042	CD8.pAbO	2297.95	2907.37	0.048
								LRIG2	0	0.22	0.042				
								MEX3A	0	0.22	0.042				
								MAGIX	0.05	0.5	0.045				
								CADM1	0	0.39	0.049				

Table S15 – the significant expressed parameters in group 1 and group 2 comparison for helper T cells.

	G1 vs. G2		G3 vs. G4		G5 vs. G6		G7 vs. G8	
**Parameters**	**Mean diff**	***p*_values**	**Mean diff**	***p*_values**	**Mean diff**	***p*_values**	**Mean diff**	***p*_values**
AL136454.1	0.18	0.031	0	1	0	1	0	1
APOBEC3G	0.09	0.044	0	1	0	1	0	1
BIRC3	0.18	0.0097	0.05	0.33	0	1	-0.06	0.33
CD247	0.18	0.0097	0	1	0	1	0	1
CD27	0.14	0.032	0	1	-0.05	0.33	0	1
CD6	0.23	0.011	0	1	0	1	0	1
CD69	0.11	0.024	0	1	0.11	0.35	0.05	0.33
CD9	-0.25	0.031	0	1	0	1	0	1
CNTLN	-0.17	0.043	0.26	0.021	-0.14	0.083	0	0.96
CXCR3	0.16	0.0066	0	1	0	1	0	1
DMXL1	0.27	0.0018	0	1	0	1	0.05	0.33
DUSP2	0.16	0.032	0	1	-0.05	0.33	0	1
DUSP4	0.23	0.0028	0	1	0	1	0	1
FCGR3A	-0.29	0.032	0	1	0	1	0	1
FCN1	-0.42	0.038	0	1	0	1	0	1
FTH1	-0.31	0.043	0	1	0	1	0	1
GDF5OS	0.14	0.013	0.05	0.33	0.11	0.35	0	1
ICOS	0.23	0.017	0	1	0	1	0	1
IFITM2	0.23	0.029	0	1	0	1	0	1
IFITM3	0.23	0.011	0	1	0	1	0	1
IFNGR1	-0.27	0.011	0	1	0	1	0	1
IKZF1	0.09	0.044	0	1	0	1	0	1
IL12RB2	0.09	0.044	0	1	0	1	0	1
IL4R	0.2	0.027	0	1	0	1	0	1
IRF4	0.34	0.0056	0	1	0	1	0	1

LAT	0.23	0.0061	0	1	0	1	0	1
LCK	0.52	0.00012	0	1	0	1	0	1
LEF1	0.41	0.00032	0	1	0	1	0	1
LY86	-0.38	0.0093	0	1	0	1	0	1
MAP4K4	0.16	0.018	0.05	0.33	-0.05	0.33	-0.61	0.077
CD3.pAbO	403.19	0.0078	127.15	0.55	24.38	0.95	-617.15	0.02
CD4.pAbO	2294.59	3.70E-16	991.45	2.20E-05	61.16	0.83	233.83	0.0012
CD127.pAbO	-1950.9	0.013	7222.57	0.00011	1775.65	0.37	-8978.69	0.0071
CD16.pAbO	-2962.49	3.50E-05	2060.31	0.00013	374.07	0.75	-2534.87	0.00016
CD197.pAbO	-2295.55	0.019	4661.22	9.80E-06	633.25	0.66	-4451.72	0.00059
CD19.pAbO	-302.11	0.014	589.31	5.20E-05	140.38	0.58	-724.8	0.00075
CD25.pAbO	2092.02	7.10E-10	2516.13	2.50E-05	413.73	0.21	-813.96	1.20E-05
CD27.pAbO	4693.67	9.30E-13	820.91	3.70E-05	645.36	0.14	-602.64	0.00033
CD45RA.pAbO	366.7	1.00E-05	270.23	0.0054	19.15	0.88	-207.3	0.0078
CD56.pAbO	-517.97	0.011	302.47	0.00078	-66.89	0.73	-1054.75	0.00084
CD8.pAbO	-820.49	0.015	904.19	7.40E-05	100.89	0.84	-1283.66	0.00022
IgD.pAbO	-416.35	0.019	450.46	3.00E-04	25	0.92	-780.46	0.00091
TCRgd.pAbO	-6317.4	0.018	15111.24	1.00E-05	2252.21	0.57	-12668.4	0.0011

Table S16 – the significant expressed parameters in group 1 and group 2 comparison for cytotoxic T cells.

	G1 vs. G2		G3 vs. G4		G5 vs. G6		G7 vs. G8	
**Parameters**	**Mean diff**	***p*_values**	**Mean diff**	***p*_values**	**Mean diff**	***p*_values**	**Mean diff**	***p*_values**
AL136454.1	0.31	0.044	0	1	0	1	0	1
BIRC3	0.29	0.03	0.05	0.33	0	1	0	1
C20orf96	0.21	0.0033	0.05	0.33	0.12	0.35	0.05	0.33
CATSPER2	-0.14	0.043	-0.07	0.34	-0.1	0.16	0	1
CBLB	0.15	0.032	0	1	0	1	0	1
CD200	0.1	0.044	0	1	0	1	0	1
CD27	0.31	0.0033	0	1	0	1	0	1
CD37	0.1	0.044	0.05	0.33	0	1	0	1
CD44	0.18	0.018	0	1	0	1	0	1
CD6	0.33	0.01	0	1	-0.05	0.33	0	1
CD8A	0.79	2.3E-06	0	1	0	1	-0.05	0.33
CD8B	0.76	0.004	0	1	0	1	0	1
CNTLN	-0.14	0.043	0.32	0.03	-0.1	0.16	0.06	0.56
CSF2	0.38	0.02	0	1	0	1	-0.05	0.33
CTSW	0.18	0.0064	0	1	0	1	0	1
CXCR3	0.23	0.011	0	1	0	1	0	1
DUSP2	0.33	0.0075	0	1	0	1	0	1
DUSP4	0.1	0.044	0	1	0	1	0	1
GDF5OS	0.15	0.032	0.26	0.056	0.07	0.58	0.11	0.16
IFITM2	0.22	0.035	0	1	0	1	0	1
IFITM3	0.18	0.018	0	1	0	1	0	1
IRF4	0.27	0.018	0	1	0	1	0	1
KCNJ3	0.13	0.023	0.16	0.19	0	1	0.06	0.56
LAT	0.28	0.014	0.11	0.33	0	1	0	1
LCK	0.24	0.021	0	1	0	1	0	1

LEF1	0.33	0.00089	0	1	0	1	0	1
LIF	0.23	0.048	0.05	0.33	0	1	0	1
MAP4K4	0.1	0.044	0.05	0.33	0.07	0.58	0.26	0.38
MCM4	0.23	0.011	0	1	0	1	-0.06	0.56
MIXL1	-0.14	0.043	0	0.99	0.12	0.35	-0.21	0.14
CD4.pAbO	-212.31	0.00014	340.94	0.13	287.48	0.49	109.84	0.27
CD127.pAbO	-1895.56	0.011	4641.6	0.036	2036.83	0.32	9598.74	0.0039
CD16.pAbO	-1671.62	0.003	1759.38	0.0051	511.83	0.67	2396.63	0.000087
CD197.pAbO	-2295.9	0.013	3410.47	0.0055	928.83	0.52	4768.95	0.000079
CD19.pAbO	-297	0.01	469.31	0.005	172.76	0.5	724.53	0.00048
CD25.pAbO	1305.85	0.000036	2283.63	1.2E-07	254.71	0.41	833.68	1.9E-07
CD27.pAbO	3952.02	1.3E-10	652.34	0.0013	468.88	0.29	424.73	0.0031
CD45RA.pAbO	705.07	3.2E-07	293.63	0.039	227.55	0.23	17.42	0.84
CD45.pAbO	1366.8	0.029	737.78	0.32	56.45	0.97	281.58	0.44
CD56.pAbO	-574.43	0.0042	430.25	0.021	47.72	0.83	880.79	0.0018
CD8.pAbO	3785.93	1.1E-09	1760.34	0.000046	458.88	0.34	609.42	0.048
IgD.pAbO	-455.46	0.0049	401.58	0.018	196.64	0.48	749.52	0.0002
TCRgd.pAbO	-6812.01	0.011	11631.32	0.0038	3428.24	0.39	13151.74	0.00034

Table S17 – the significant expressed parameters in group 1 and group 2 comparison for B cells.

Parameters	G1 vs. G2			G3 vs. G4			G5 vs. G6			G7 vs. G8		
	G1_mean	G2_mean	*p*_values	G3_mean	G4_mean	*p*_values	G5_mean	G6_mean	*p*_values	G7_mean	G8_mean	*p*_values
AC0036881.1	0.15	0	0.043	0.08	0.1	0.86	0	0	1	0	0.06	0.33
ADA	0	0.14	0.043	0	0	1	0	0	1	0	0	1
CNTLN	0.15	0	0.043	0	0.25	0.056	0.05	0	0.33	0.17	0.18	0.95
ICOS	0	0.15	0.043	0	0	1	0	0	1	0	0	1
IFNGR1	0.19	0	0.022	0	0	1	0	0	1	0	0	1
LY86	0.15	0	0.043	0	0	1	0	0	1	0	0	1
MIXL1	0.15	0	0.043	0.08	0.2	0.48	0	0.33	0.081	0.17	0.18	0.96
CD4.pAbO	323.0	1319.7	0.0026	468.6	1288.1	0.0064	854.7	1004.8	0.67	546.3	513.1	1.75
CD25.pAbO	469.3	14.92	0.00014	687.6	3195.1	6.8E-06	1094.5	1687.7	0.11	790.0	1527.9	2.4E-08
CD27.pAbO	538.6	6089.3	0.00036	723.7	1546.3	6.8E-06	1089.3	1611.4	0.17	777.4	02064.2	0.0065
CD45RA.pAbO	251	539.0	0.0066	219.9	546.8	0.00048	796.3	800.2	0.98	213.2	224.2	0.83

Table S18 – the significant expressed parameters in group 1 and group 2 comparison for NK/T cells.

Parameters	G1 vs. G2			G3 vs. G4			G5 vs. G6			G7 vs. G8		
	**G1_mean**	**G2_mean**	***p*_values**	**G3_mean**	**G4_mean**	***p*_values**	**G5_mean**	**G6_mean**	***p*_values**	**G7_mean**	**G8_mean**	***p*_values**
CCL2	0.38	0	0.017	0	0	1	0	0	1	0	0	1
CD14	0.19	0	0.042	0	0	1	0	0	1	0	0	1
CNTLN1	0.19	0	0.042	0	0.2	0.17	0.18	0	0.17	0.09	0.07	0.83
FCGR3A	0.52	0.12	0.037	0	0	0	0	0	1	0	0	1
FCN1	0.38	0	0.0022	0	0	0	0	0	1	0	0	1
IFNGR11	0.33	0	0.0049	0	0	0	0	0	1	0	0	1
ITGAX	0.33	0	0.0049	0	0	0	0	0	1	0	0.13	0.33
LILRB4	0.29	0	0.03	0	0	0	0	0	1	0	0	1
LY861	0.33	0	0.031	0	0	0	0	0	1	0	0	1

Table S19 – Comparative approach considering condition G1 vs. G2 as gold standard and define differentially expressed markers for each cell type subset indicating the fraction of recovered differentially expressed genes in experimental conditions (G3 vs G4, G5 vs G6, and G7 vs G8) comparison with the gold standard.

B cells				NK/T cells				Helper T cells				Cytotoxic T cells			
**Significant marker G1 vs. G2**	**G3 vs.G4**	**G5 vs.G6**	**G7 vs. G8**	**Significant marker G1 vs. G2**	**G3 vs.G4**	**G5 vs.G6**	**G7 vs. G8**	**Significant marker G1 vs. G2**	**G3 vs.G4**	**G5 vs.G6**	**G7 vs. G8**	**Significant marker G1 vs. G2**	**G3 vs.G4**	**G5 vs.G6**	**G7 vs. G8**
CD25. pAbO			Yes	FCN1				CD4.pAbO	Yes		Yes	CD27. pAbO	Yes		Yes
CD27. pAbO	Yes		Yes	IFNGR1				CD27.pAbO			Yes	CD8. pAbO	Yes		Yes
CD45RA. pAbO				ITGAX				CD25. pAbO	Yes		Yes	CD45RA. pAbO	Yes		
IFNGR1				CCL2				CD45RA. pAbO			Yes	CD8A			
AC003681.1				LILRB4				CD16. pAbO	Yes		Yes	CD25. pAbO	Yes		Yes
ADA				LY86				LCK				CD4. pAbO			
CNTLN				FCGR3A				LEF1				LEF1			
ICOS				CD14				DMXL1				CD16. pAbO	Yes		Yes
LY86				CNTLN				DUSP4				C20orf96			
MIXL1								IRF4				CD27			
								LAT				CD8B			
								CXCR3				CD56.p AbO	Yes		Yes

CD3. pAbO				CTSW		Yes	
LY86				DUSP2			
BIRC3				CD6			
CD247				CXCR3			
CD6				MCM4			
IFITM3				CD127. pAbO	Yes		Yes
IFNGR1				TCRgd. pAbO	Yes		Yes
CD56. pAbO	Yes		Yes	CD197. pAbO			Yes
GDF5OS				LAT			
CD127. pAbO	Yes		Yes	CD44			
CD8. pAbO	Yes		Yes	IFITM3			
ICOS				IRF4			
MAP4K4				CSF2			
TCRgd.pAbO	Yes		Yes	LCK			
CD197. pAbO			Yes	KCNJ3			
CD69				CD45.pAbO			
IL4R				BIRC3			

		IFITM2				CBLB			
		AL136454.1				GDF5OS			
		CD9				IFITM2			
		CD27				CATSPER2			
		DUSP2				CNTLN	Yes		
		FCGR3A				MIXL1			
		FCN1				AL136454.1			
		CNTLN	Yes			CD200			
		FTH1				CD37			
		APOBEC3G				DUSP4			
		IKZF1				MAP4K4			
		IL12RB2				LIF			

Figure S1– Quality controls for single-cell multi-omics data generated on selected permeabilization/fixation methods on lymphocytes (A) Comparison of the distribution number of qualified cells within the HiSeqX and NextSeq cell showing the more balanced pattern in the HiSeqX platform with higher read depth based on proteomics data. (B) Comparison of the distribution number of qualified cells and their transcriptome density within different experimental conditions analysis using HiSeqX data output. (C) Top 100 expressed genes were affected by fixation/permeabilization within different experimental conditions analysis. G1: Unstimulated PBMCs, G2: Stimulated PBMCs, G3: Unstimulated PBMCs with fixation, G4: Stimulated PBMCs with fixation, G5: Unstimulated PBMCs with Fixation/Permeabilization method 1, G6: Stimulated PBMCs with Fixation/Permeabilization method 1, G7: Unstimulated PBMCs with Fixation/Permeabilization method 2, G8: Stimulated PBMCs with Fixation/Permeabilization method 2.

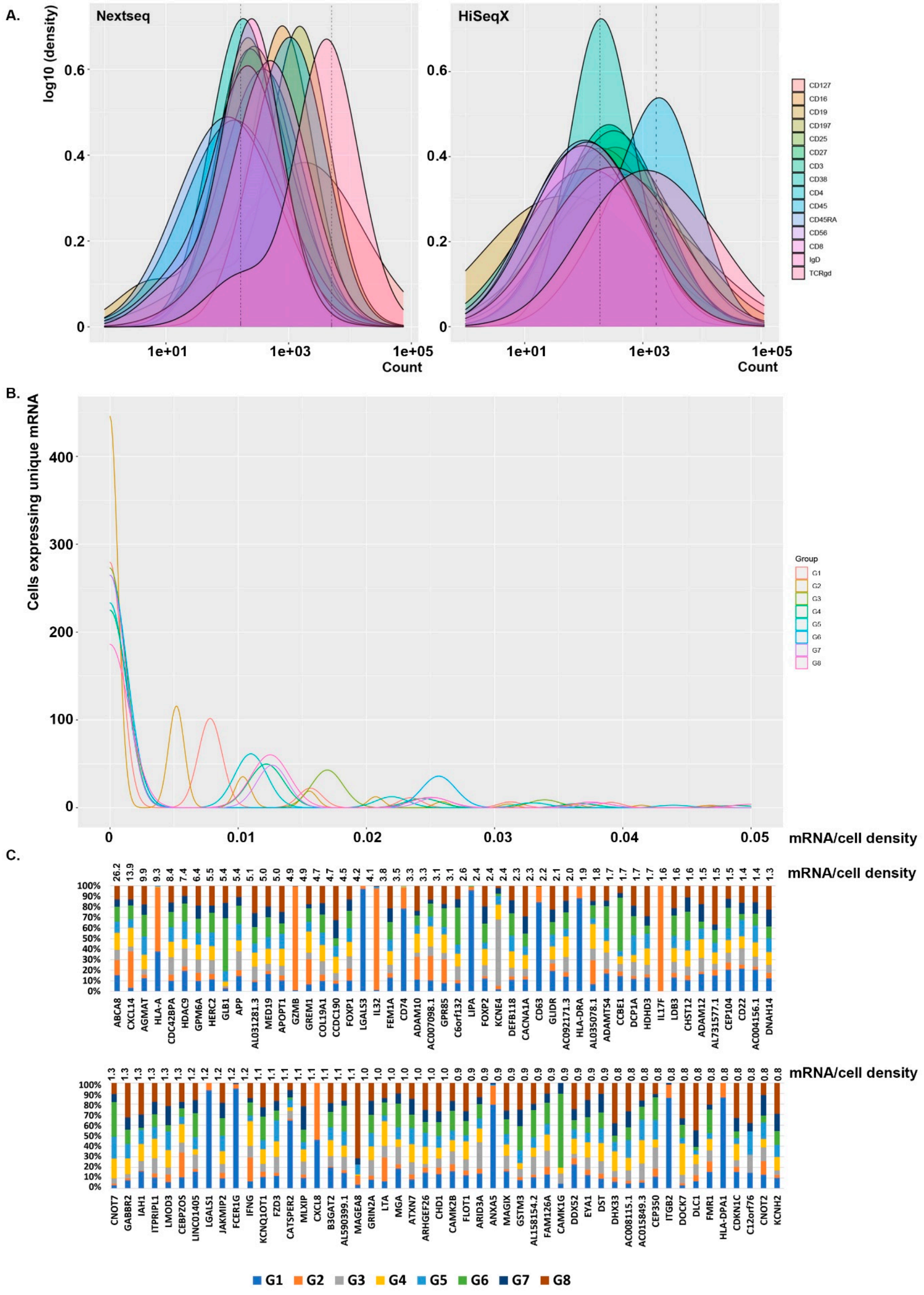

Figure S2 – Visualizations of the top significant parameters in each cell type while comparing group 1 and group 2.

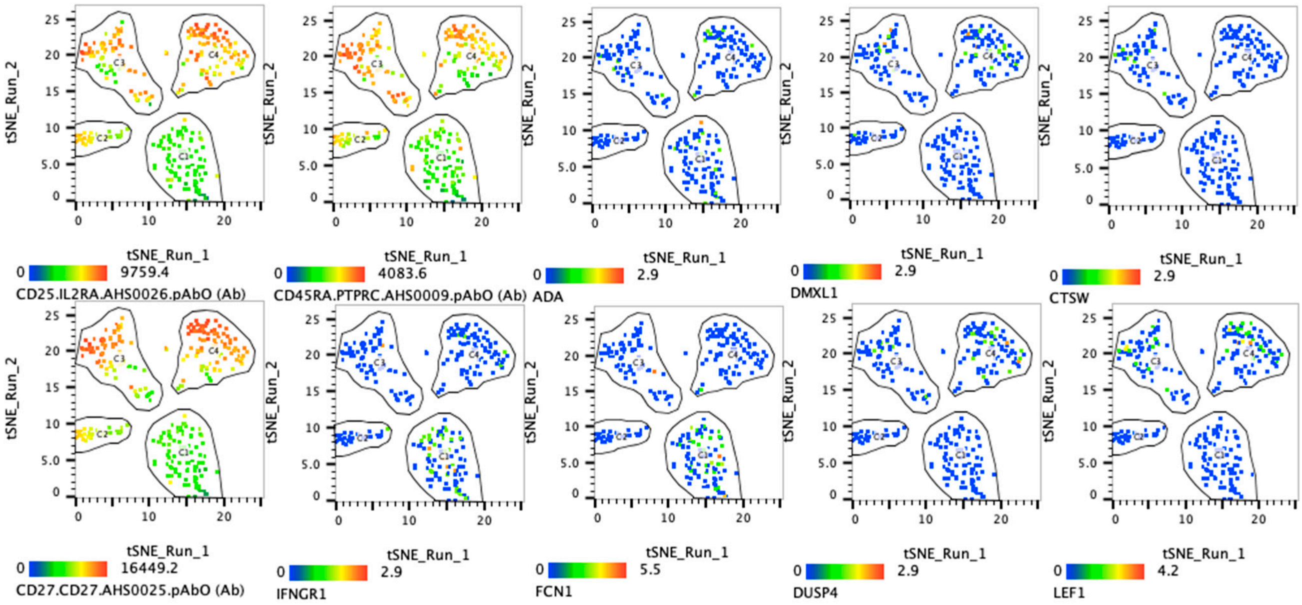

Figure S3 – Volcano plots of comparing differential expression of cluster 1 vs. cluster 2 (C1 vs C2), C1 vs C3, C1 vs C4.

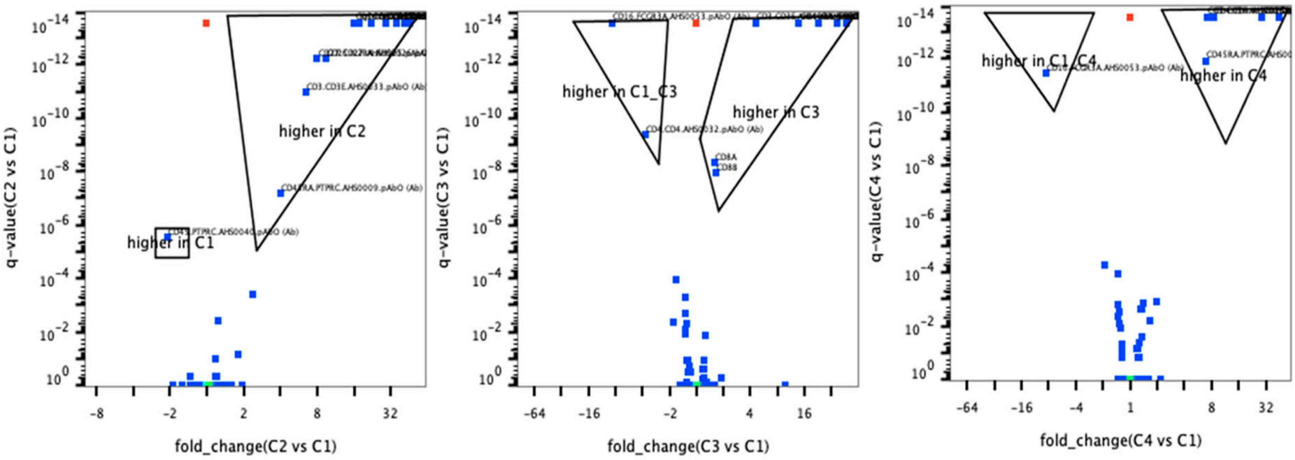

C1 vs C2	
higher in C1	higher in C2
CD45.pAbO (Ab)	CD3.pAbO
	CD127.pAbO (Ab)
	CD197.pAbO (Ab)
	CD19.pAbO (Ab)
	CD25.pAbO (Ab)
	CD27.pAbO (Ab)
	CD45RA.pAbO (Ab)
	CD56.pAbO (Ab)
	CD8.pAbO (Ab)
	IgD.pAbO (Ab)
	TCRgd.pAbO (Ab)

C1 vs C3	
higher in C1	higher in C3
CD4.pAbO (Ab)	CD3.pAbO
CD16.pAbO (Ab)	CD25.pAbO (Ab)
	CD27.pAbO (Ab)
	CD45RA.pAbO (Ab)
	CD8.pAbO (Ab)
	CD8A
	CD8B

C1 vs C4	
higher in C1	higher in C4
CD16.pAbO (Ab)	CD3.pAbO
	CD4.pAbO (Ab)
	CD25.pAbO (Ab)
	CD27.pAbO (Ab)
	CD45RA.pAbO (Ab)

Figure S4- T-SNE plot of experimental conditions 1 and 3 based on the transformed expression of the 6 lineage markers in the cells from the PBMC dataset to detect the impact of fixation.

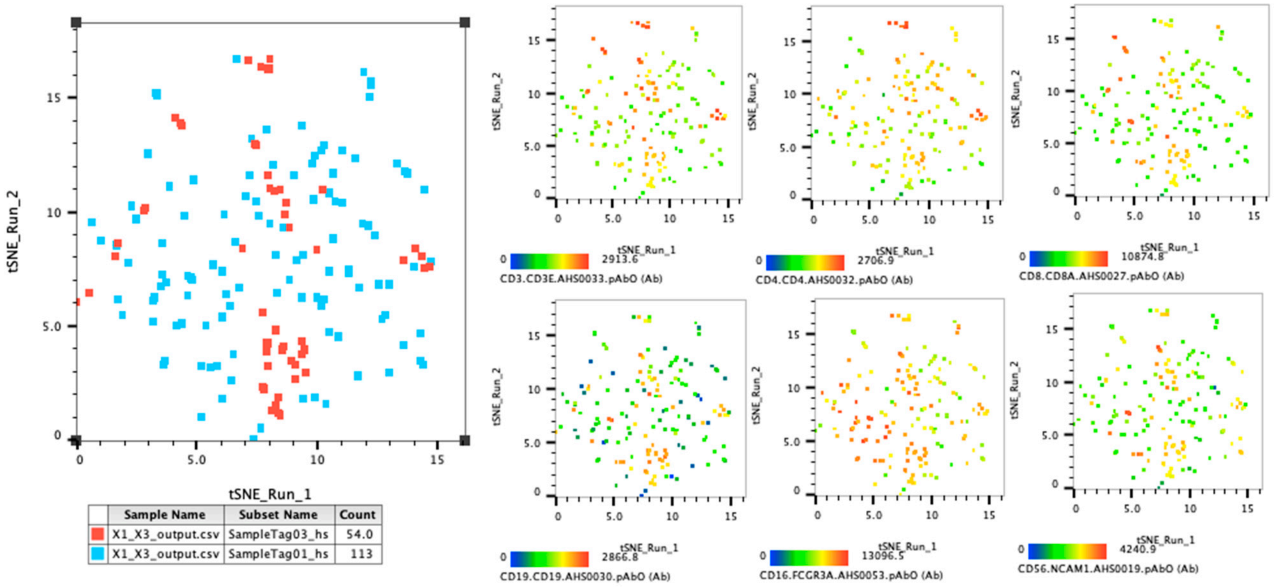

Figure S5- T-SNE plot of experimental conditions 1 and 5 based on the transformed expression of the 6 lineage markers in the cells from the PBMC dataset to detect the impact of permeabilization method 1 and fixation.

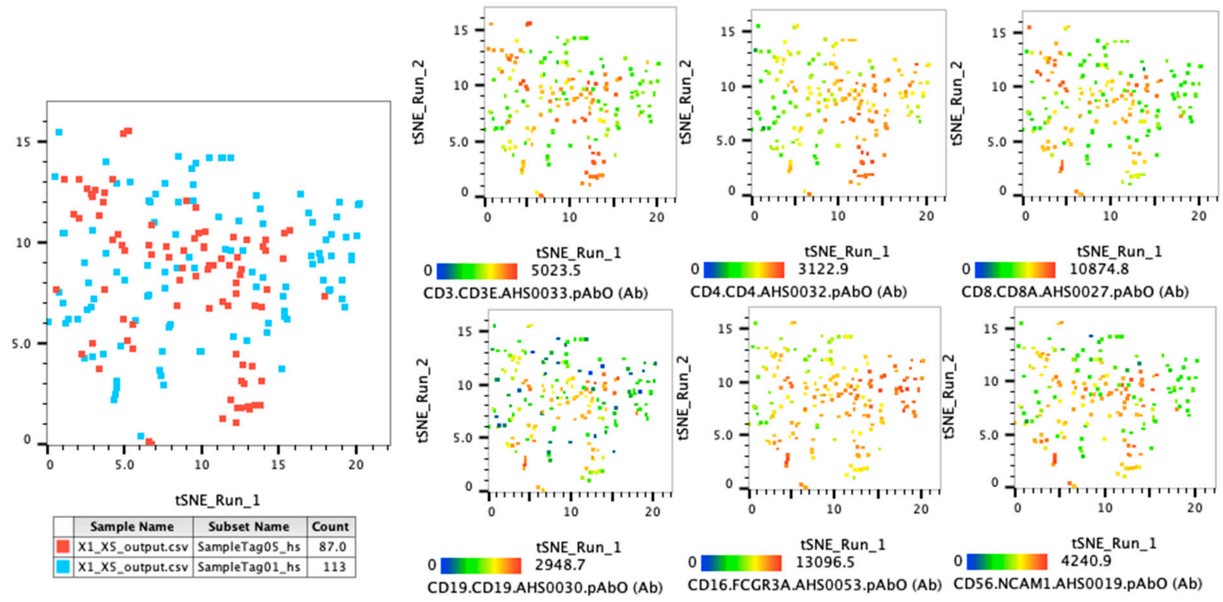

Figure S6- T-SNE plot of experimental conditions 1 and 7 based on the transformed expression of the 6 lineage markers in the cells from the PBMC dataset to detect the impact of permeabilization method 2 and fixation.

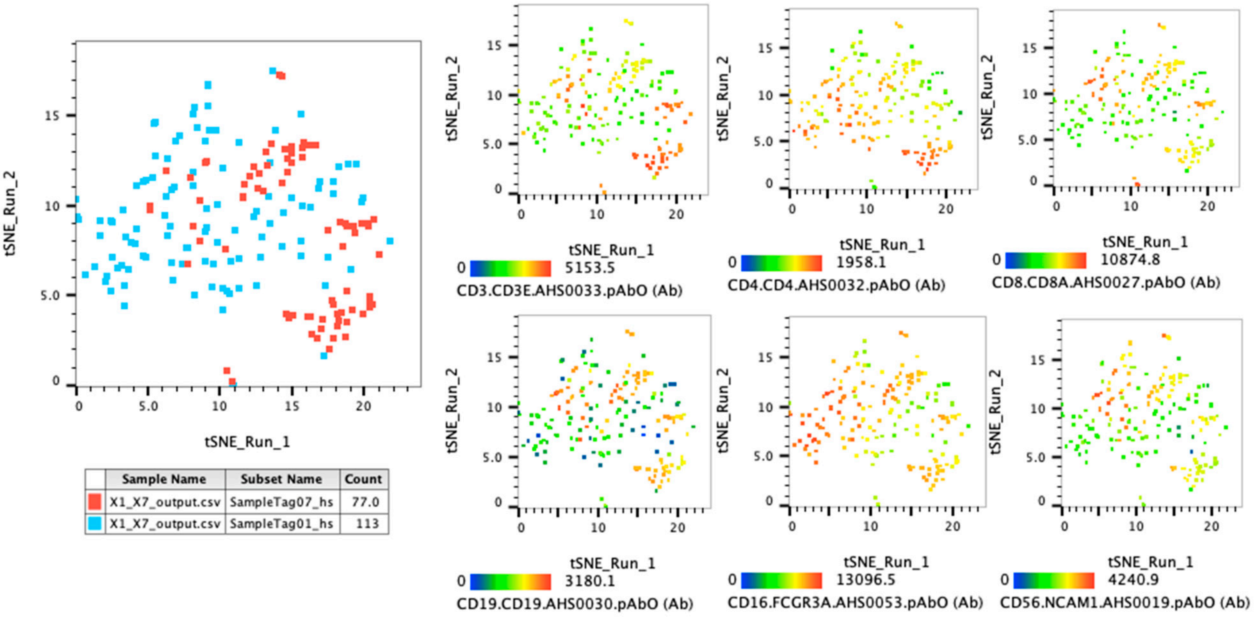

Figure S7 – Visualizations of the top significant parameters in each cell type while comparing group 1 and group 3.

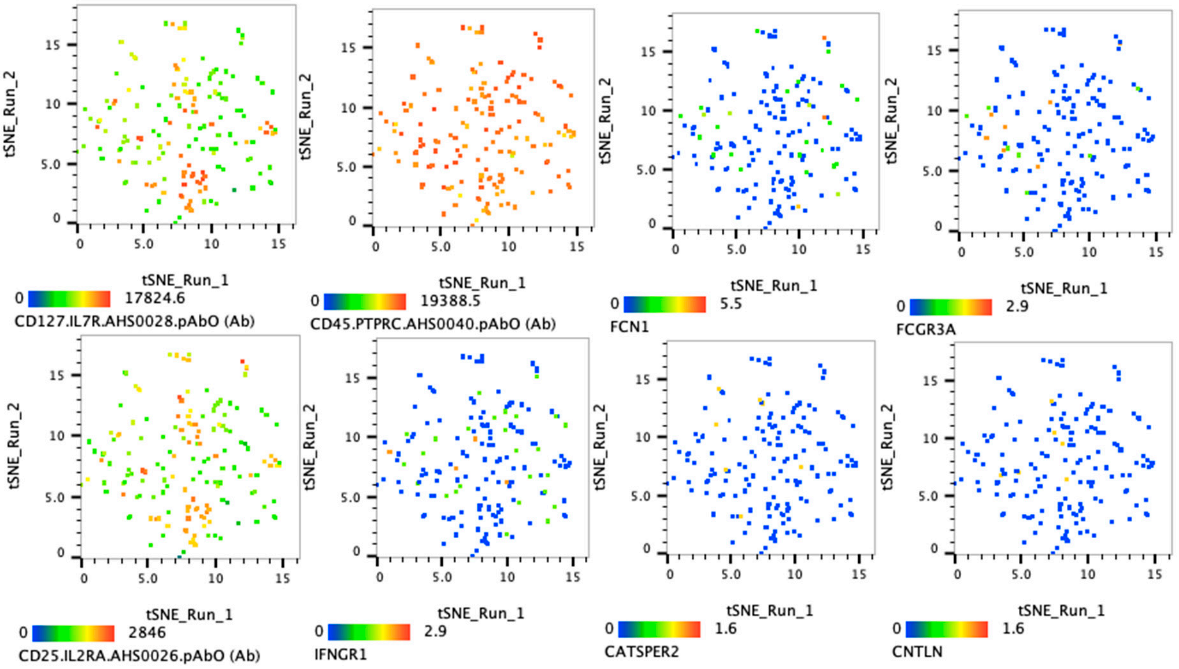

Figure S8 – Visualizations of the top significant parameters in each cell type while comparing group 1 and group 5.

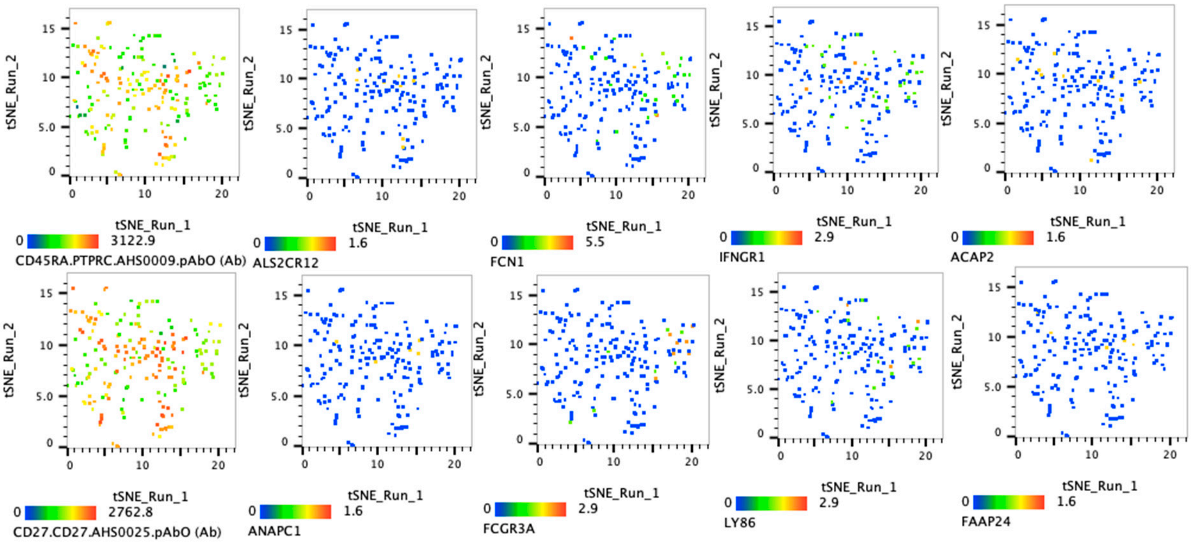

Figure S9 – Visualizations of the top significant parameters in each cell type while comparing group 1 and group 7.

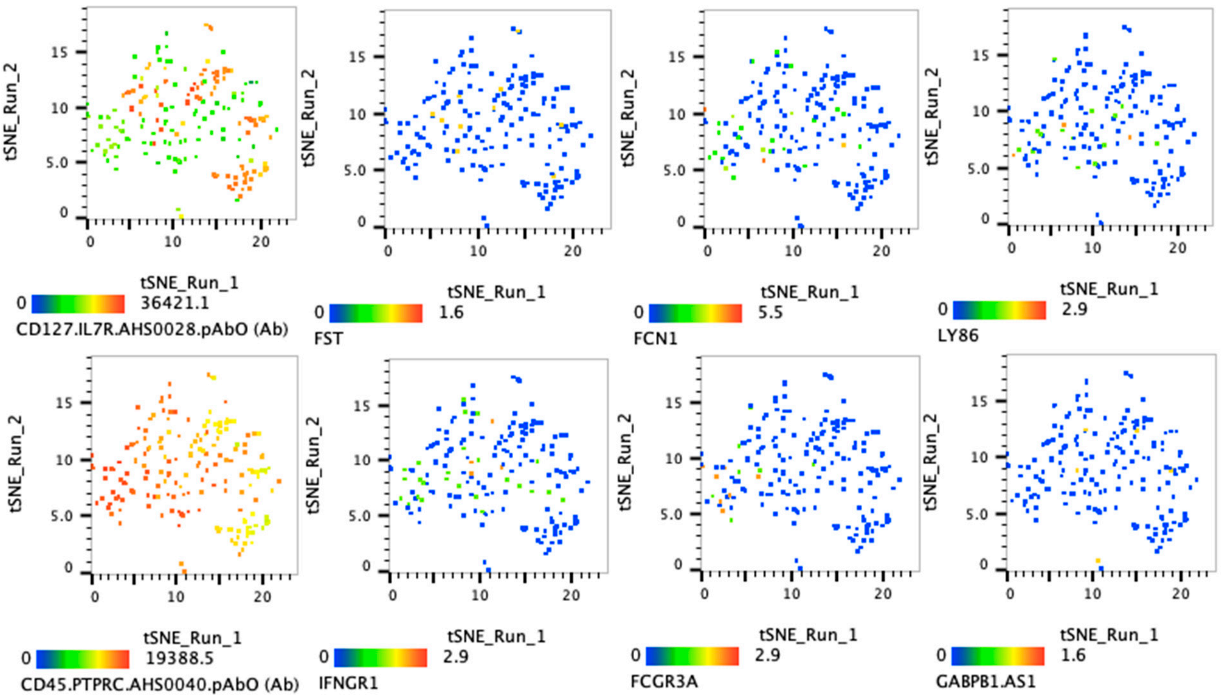

Figure S10- T-SNE plot of experimental conditions 3 and 4 based on the transformed expression of the 6 lineage markers in the cells from the PBMC dataset to detect the impact of stimulation.

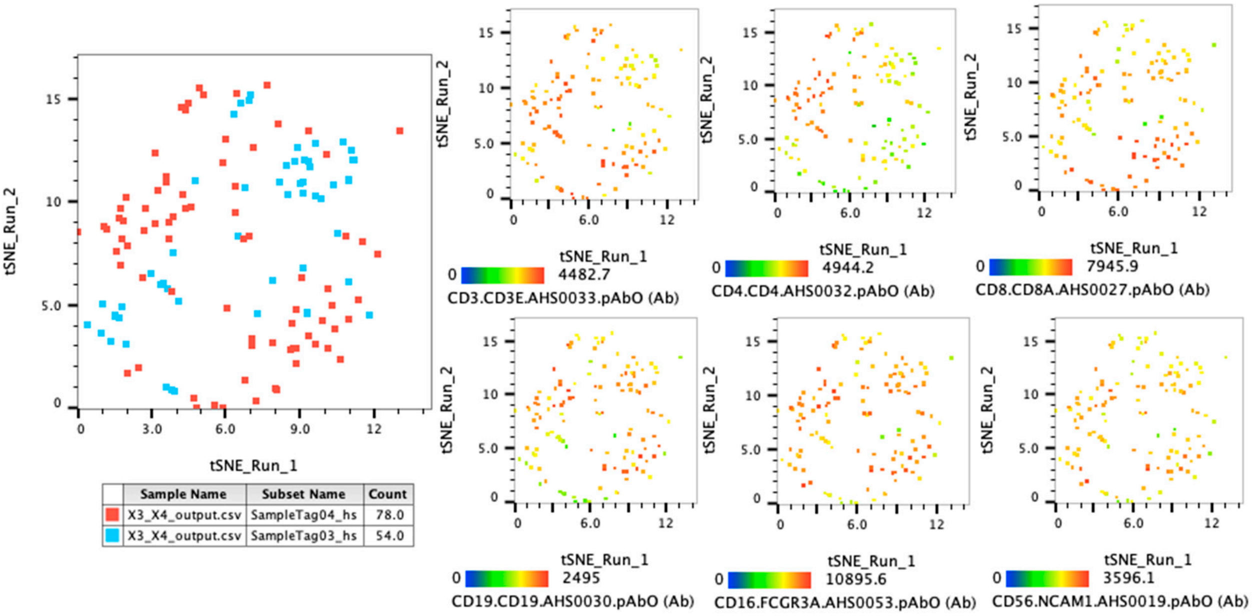

Figure S11 – Visualizations of the top significant parameters in each cell type while comparing group 3 and group 4.

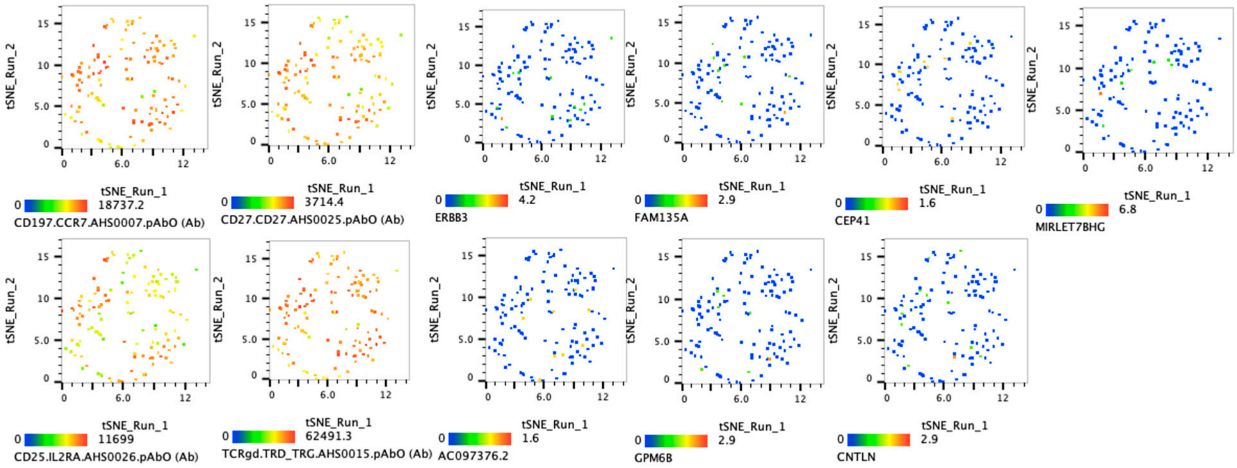

Figure S12- T-SNE plot of experimental conditions 5 and 6 based on the transformed expression of the 6 lineage markers in the cells from the PBMC dataset to detect the impact of stimulation.

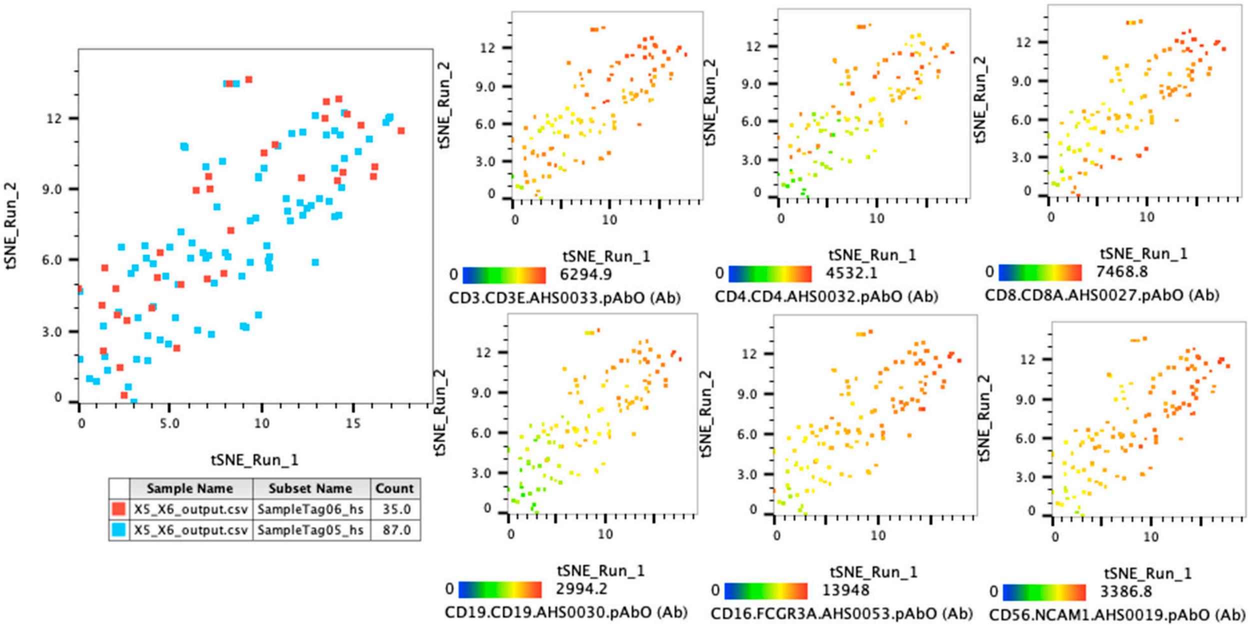

Figure S13 – Visualizations of the top significant parameters in each cell type while comparing group 5 and group 6.

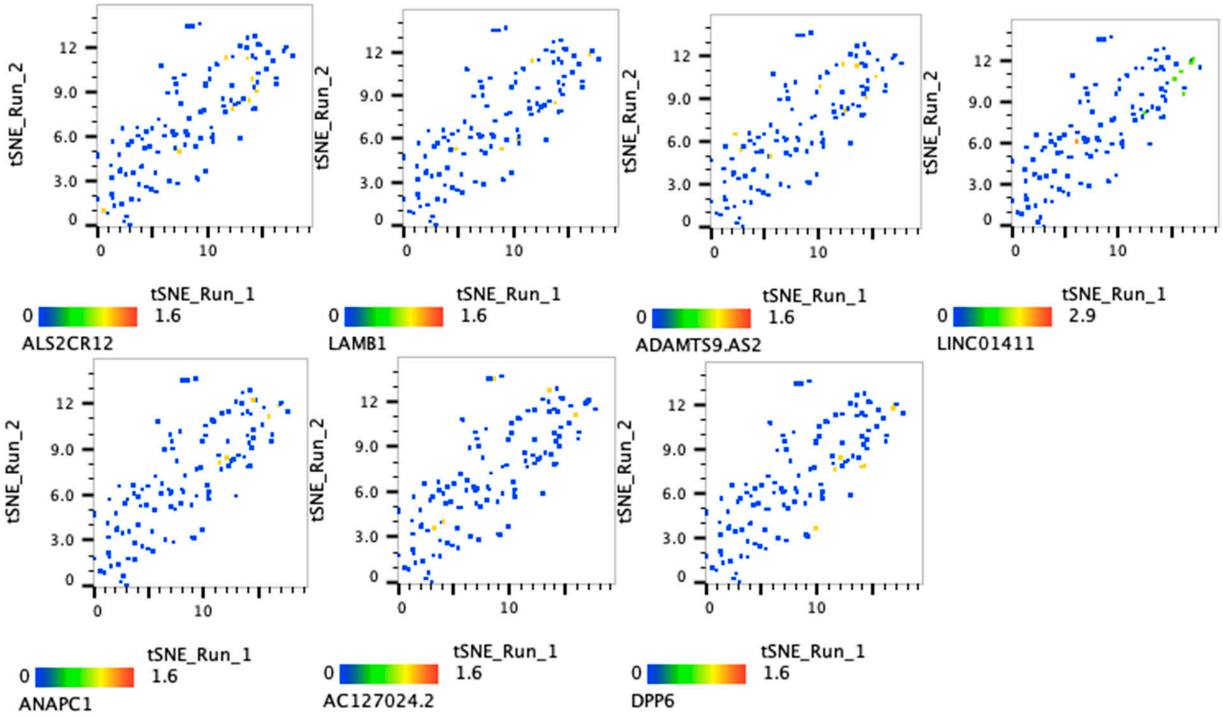

Figure S14- T-SNE plot of experimental conditions 7 and 8 based on the transformed expression of the 6 lineage markers in the cells from the PBMC dataset to detect the impact of stimulation.

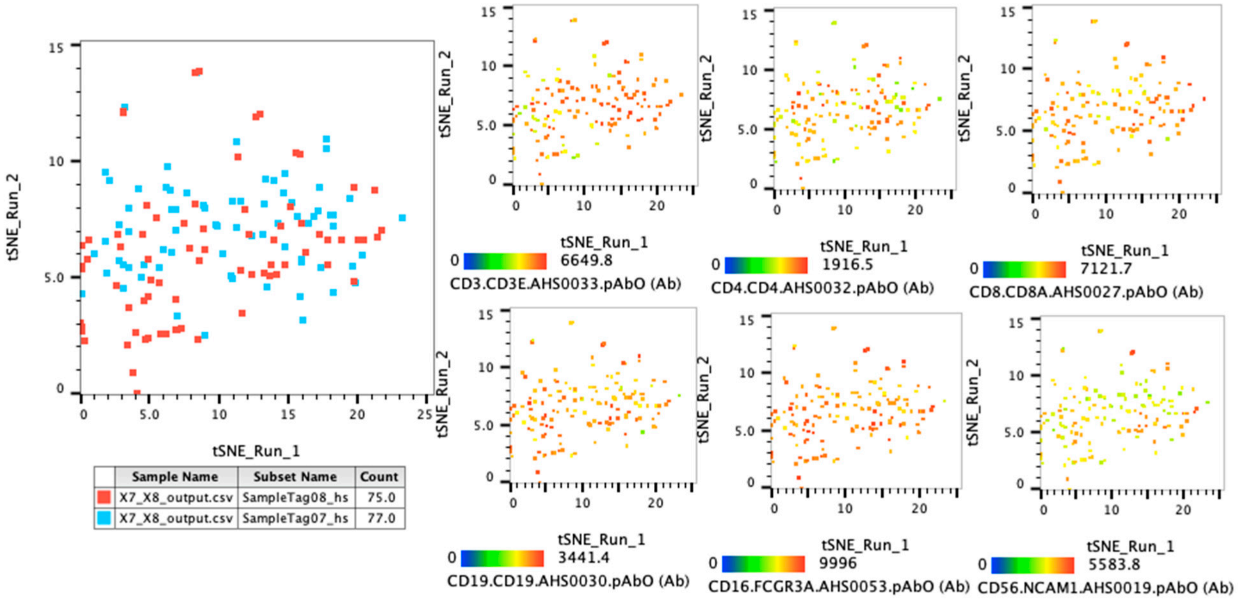

Figure S15 – Visualizations of the top significant parameters in each cell type while comparing group 7 and group 8.

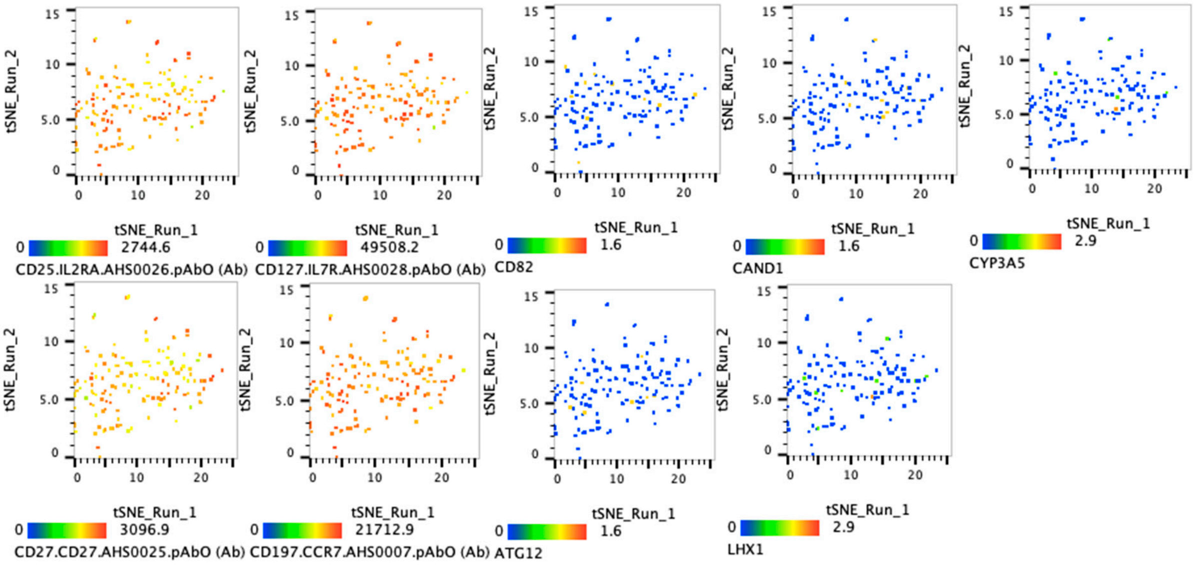

Supplement: Supplementary file 1 [file antibodies-14-00015-s001.zip › antibodies-3148476-supplementary.pdf]
